# Determination of the Preferred Stereoisomer of Natural Product Bisabolol in Chloroform Solution through Quantum Chemical Calculations of ^1^H NMR Chemical Shifts

**DOI:** 10.1021/acsomega.5c07923

**Published:** 2025-10-13

**Authors:** Haroldo C. Da Silva, Lucas H. Martorano, Fernando M. Dos Santos, Wagner B. De Almeida

**Affiliations:** † Programa de Pós-Graduação em Química (PPGQ), Instituto de Química, 28130 Universidade do Estado do Rio de Janeiro (UERJ), Maracana, Rio de Janeiro 20550-013, Brazil; ‡ Laboratório de Química Computacional e Modelagem Molecular (LQC-MM), Departamento de Química Inorgânica, Instituto de Química, 28110 Universidade Federal Fluminense (UFF), Outeiro de São João Batista s/n, Campus do Valonguinho, Centro, Niterói 24040-141, Brazil; § Laboratório de Química Teórica e Simulação Molecular (LQTSM), Departamento de Físico-Química, Instituto de Química, Pavilhão Haroldo Lisboa da Cunha, 28130 Universidade do Estado do Rio de Janeiro (UERJ), Rua São Francisco Xavier, 524, Maracana, 20550-013 Rio de Janeiro, Brazil; ∥ Departamento de Química Orgânica, Instituto de Química, 28110 Universidade Federal Fluminense (UFF), Outeiro de São João Batista s/n, Campus do Valonguinho, 24020-141, Centro, Niterói 24040-141, Brazil

## Abstract

The elucidation of natural product structures and the differentiation of stereoisomers are important issues in organic chemistry. An example is the sesquiterpene (−)-α-bisabolol (αBis), having two stereogenic centers, αBis and a flexible side chain that generates high conformational freedom. Recently, new tools named DP4+ and ANN-PRA, which are probabilistic approaches, were used in combination to solve the relative configuration of αBis (α and epi-α diastereomers). Nuclear magnetic resonance (NMR) chemical shifts obtained from density functional theory (DFT) calculations in the vacuum were used by the DP4+ and ANN-PRA computational algorithms averaged by the Boltzmann population. Although such a procedure can provide an indication of the most probable enantiomer, no information on the spatial arrangement of the preferred molecular structure present in the NMR experiment (in CDCl_3_) could be obtained. In this work, we used the DFT methodology and the polarizable continuum model approach, with the inclusion of explicit CHCl_3_ solvent molecules, to calculate ^1^H NMR spectra for various distinct trial molecular structures of αBis, encompassing α and epimeric forms, varying relevant torsion angles to find plausible minimum energy structures on the potential energy surface, including solvent effects. Through comparison between the experimental and theoretical ^1^H NMR profiles in chloroform solution, we were able to unambiguously elucidate the predominant molecular structure (enantiomer α) that reproduced faithfully the experimental ^1^H NMR pattern. This could not be done in previous work employing the DP4+ and ANN-PRA tools; however, there is an agreement that the α stereoisomer should be predominant. The preferred α-Bis molecular structure reported here will most probably interact with biological targets.

## Graphical abstract

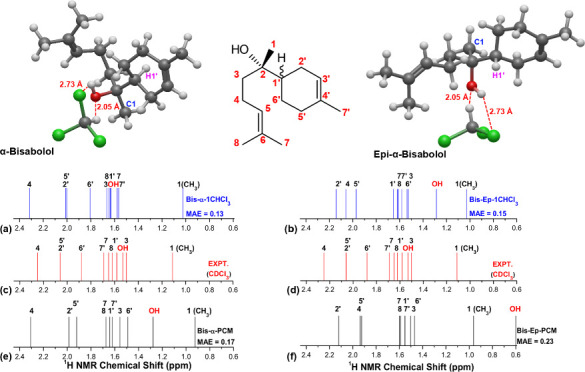

## Introduction

The determination of the molecular structure of chemical compounds in solution presenting chiral centers is a challenge in organic chemistry, in particular, in the area of natural products. A combined use of experimental nuclear magnetic resonance (NMR) data and quantum chemical calculations of NMR chemical shifts, including solvent effects, has been shown to be of great value in elucidating the structures of organic molecules in solution.^1^ However, the determination of the relative configuration of chiral molecules is not a trivial task. In the specific case of the antibiotic azithromycin (AZM), it could not be done through the analysis of experimental and theoretical ^1^H -NMR spectra.^2^ Some years ago, a method named DP4 (Diastereomeric Parameter 4) was developed by Smith and Goodman^3^ which can be used to assign the stereochemistry when only one set of NMR experimental data is available. This methodology was improved by Sarotti and collaborators,^4^ and the new version named DP4+ has stood out as one of the leading toolboxes in structural elucidation with computational NMR methods of more than 200 natural and synthetic products. The goal of the method is the probability (P_i_) of finding the correct candidate structure (i) among many possible isomers, obtained through Bayes′s theorem. It is based on the fact that the errors between experimental and calculated chemical shifts for a set of organic molecules obey a distribution defined by three terms: mean, standard deviation, and degrees of freedom.^4^ Both ^1^H and ^13^C NMR data can be used in the DP4+ scheme. A critical review on the use of DP4+ in the structural elucidation of natural products, showing advantages and limitations, was recently published.^5^

The DP4+ and artificial neural networks (ANNs)-pattern recognition analysis (PRA)^6^ tools, which are based respectively on Bayesian probability and ANNs, can be used together to solve the relative configuration of organic compounds. This methodology was applied to the analysis of the stereostructure of the sesquiterpene (−)­α-bisabolol (αBis).^7^ αBis is a natural monocyclic sesquiterpene alcohol and is known to have anti-irritant, anti-inflammatory, and antimicrobial properties, besides being used in cosmetics because of its skin-healing properties. However, αBis presents some structural characteristics that make its stereochemical analysis considerably difficult. In addition to having a flexible side chain that generates high conformational freedom, its structure has two stereogenic centers. Due to these structural features, even in combination with nuclear Overhauser effect measurements, determining the relative configuration of αBis can be a difficult assignment that can easily lead to ambiguous results.^7^ Therefore, differentiating the possible stereoisomers of this molecule, the epimers αBis and epi-αBis, is not an easy task. In a previous study,^7^ Density functional theory (DFT)^8^ calculations of NMR chemical shifts were carried out for 20 vacuum optimized structures of the αBis and its epimer epi-αBis, generating theoretical NMR chemical shift values that, alongside experimental data, were used to feed the DP4+ and ANN-PRA computational algorithms averaged by a Boltzmann population.^7^ Although by using this procedure it was possible to discriminate with a high level of confidence the correct stereoisomer of the natural product,^7^ no information regarding the spatial arrangement of the preferred molecular structure present in the NMR experiment (in CDCl_3_) could be obtained.

Therefore, we decided to tackle this problem of finding the correct configuration of αBis in chloroform solution using a standard quantum chemical procedure with the aid of DFT methodology, i.e., optimizing geometries, including solvent effects, calculating ^1^H NMR chemical shifts for each plausible molecular structure located on the potential energy surface (PES), and directly comparing the experimental (in CDCl_3_) and theoretical ^1^H NMR spectra. We believe that the best match between experimental and theoretical NMR profiles is a strong indication of the preferred molecular structure to be observed in solution, and it seems to work better than the analysis of statistical indices and relative DFT calculated thermodynamic data for a series of candidate molecules. Our results show that the sensitivity of the ^1^H NMR spectrum is much more revealing than statistical indices, making the comparative analysis of experimental and theoretical NMR spectra a very adequate procedure for structural elucidation of organic compounds and an alternative to statistical methods. It should be mentioned that in the case of the bisabolol molecule, where the experimental ^1^H NMR spectrum (in CDCl_3_) shows a regular pattern with well-spaced signals, we succeeded in predicting the preferred stereoisomer (α form) in solution using the polarizable continuum model (PCM),^9^ including explicit CHCl_3_ solvent molecules in DFT calculations of NMR chemical shifts. However, for larger organic molecules, as for example, azithromycin,^2^ the ^1^H NMR spectrum can be too crowded not allowing a precise assignment of each NMR signal, and in such case, the DP4+ and ANN-PRA computational algorithms seem more adequate since they are not based on the analysis of individual NMR peaks.

## Calculations

Random structures of α and epimeric forms of bisabolol (Scheme 1) were built using GaussView^10^ (the same software used to generate the representations shown in this paper), and then the geometries were fully optimized (using the Gaussian 09 package, as in all other quantum chemical calculations^11^) at the DFT level of theory^8^ using the ωB97X-D functional^12^ and 6-31G­(d,p) basis set,^13^ and the PCM model^9^ to describe solvent effects (chloroform solvent). These two optimized structures were used as starting points to search for other plausible minimum energy structures on the PES through a scan procedure by rotations around single bonds (using GaussView5) defined by relevant torsion angles indicated in Scheme 1. Ten distinct true minimum energy structures (all harmonic frequencies are real) were located on the PES for bisabolol, which were used as input for NMR calculations of shielding constants (σ), with chemical shifts (δ) determined on a δ-scale relative to tetramethylsilane (TMS) as an internal reference, using the gauge-independent atomic orbital (GIAO) method,^14^ employing the B3LYP functional^15,16^ and 6-31G­(d,p) basis set^13^ plus inclusion of solvent effects using implicit (PCM)^9^ and explicit solvent models. The reason for the choice of the two functionals was already mentioned in previous work,^1^ with ωB97X-D being adequate for predicting structural and relative energy data while B3LYP is more suitable for calculating NMR chemical shifts.

**Scheme 1:**
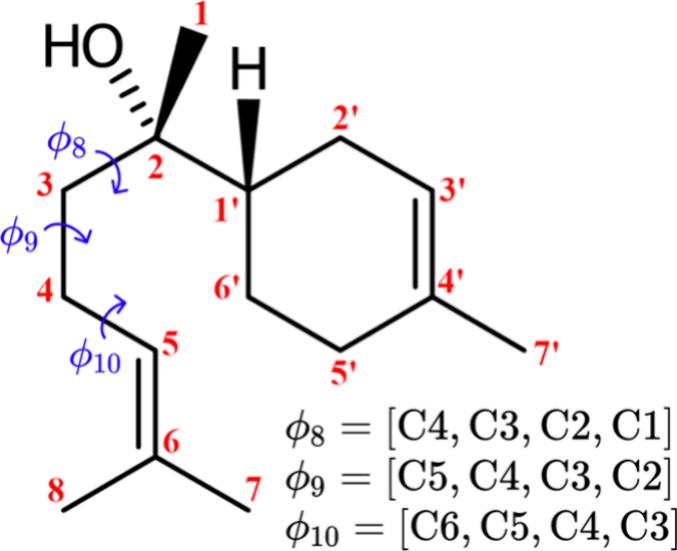
Structure and Numbering Scheme of α-Bis, Including Definition of Selected Torsion Angles Used for SCAN Purpose[Fn sch1-fn1]

a
**H1**: CH_3_; **H3**: CH_2_; **H4**: CH_2_; **H5**: CH; **H7**: CH_3_; **H8**: CH_3_; **H1’**: CH; **H2’**: CH_2_; **H3′**: CH; **H4’**: CH_3_; **H5′**: CH_2_; **H6’**: CH_2_.

## Results and Discussion

Relevant torsion angles for ten distinct minimum energy structures located on the PES for bisabolol (named structures **I** to **X**) optimized at the ωB97X-D/6-31G­(d,p)-PCM-Chloroform level of theory are given in Table 1. The **ϕ**_**3**_ and **ϕ**_**4**_ angles refer to the stereogenic centers **H1’** (bound to C1’) and **C2,** respectively, while **ϕ**_**8**_, **ϕ**_**9**_, and **ϕ**_**10**_ are connected to the side chain carbons. It can be seen from the torsion angle values reported in Table 1 that all ten structures are very distinct, and they can be considered a reasonable sample of the plausible molecular structures to be present in chloroform solution. The signs of **ϕ**_**3**_ and **ϕ**_**4**_ angles indicate the relative positions of carbon **C2** and hydrogen atom (**H1’**), respectively, defining the alpha and epimeric forms of bisabolol. It can be seen that the torsion angle related to the OH group (**ϕ**_**11**_) does not show a remarkable variation (changing sign, for example) among the ten different conformations.

**Table 1:** ωB97X-D/6-31G­(d,p)-PCM Optimized Selected Torsion Angles (**ϕ**_**i**_/°) for α-Bisabol (**Str-I**, **Str-III**, **Str-V**, **Str-VII**, **Str-IX**) and Its Epimeric Form (**Str-II**, **Str-IV**, **Str-VI**, **Str-VIII**, **Str-X**)­^a^ ^,^ ^b^

strs.	ϕ_1_	ϕ_2_	ϕ_3_	ϕ_4_	ϕ_5_	ϕ_6_	ϕ_7_	ϕ_8_	ϕ_9_	ϕ_10_	ϕ_11_
**I**	179.9	–168	16.3	74.3	–56.8	59.0	179.9	52.6	165.7	–80.9	66.5
**II**	175.3	176.2	–13.6	–67.3	–62.1	54.2	175.3	–53.1	–178.7	–87.7	51.5
**III**	–149.9	–172.1	15.3	69.4	–30.4	85.8	–149.9	–58.4	–64.6	138.8	69.3
**IV**	175.4	174.7	–14.8	–69.1	–61.5	54.7	175.4	–52.9	–173	102.3	51.1
**V**	–173.5	–168.5	16.1	74.1	–52.6	63.3	–175.5	175.8	159.0	–77.5	62.4
**VI**	163.1	173.5	–14.2	–69.5	–72.2	44.3	163.1	178.8	160.5	–81.6	56.7
**VII**	–179.0	–166.6	16.8	76.7	–58.1	57.1	–179.0	–97.4	–178.6	–100.9	60.1
**VIII**	178.2	178.3	–12.0	–66.9	–55.1	59.8	178.2	92.6	–172.1	–86.9	60.7
**IX**	–151.6	–173.2	14.1	68.5	–31.7	84.5	–151.6	–55.9	–173.7	–83.9	63.4
**X**	152.2	178.3	–14.0	–68.8	–81.6	34.2	152.2	60.3	165.9	–80.0	62.5

a No explicit solvent molecules were included.

b **ϕ**_**1**_: [C2’.C1’.C2.C3]; **ϕ**_**2**_: [C3′.C2’.C1’.C2]; **ϕ**_**3**_: [C6’.C5′.C4’.C3′]; **ϕ**_**4**_ **: [H,C1’.C2’.C3′]; ϕ**_**5**_ **: [C1.C2.C1’.C2’]; ϕ**_**6**_: **[O.C2.C1’.C2’] ; ϕ**_**7**_: [C3.C2.C1’.C2’] ; **ϕ**_**8**_: [C4.C3.C2.C1’]; **ϕ**_**9**_: [C5.C4.C3.C2]; **ϕ**_**10**_: [C6.C5.C4.C3]; **ϕ**_**11**_ ;: [H,O,C2.C1’].

ωB97X-D/6-31G­(d,p)-PCM-Chloroform optimized structures for ten local minima located on the PES for bisabolol (alpha and epimeric forms) are shown in Figure 1. The very dissimilar spatial orientations can be easily seen. These are unique structures that can be considered suitable for a conformational analysis, since they were obtained through a scan procedure rotating the **ϕ**_**8**_, **ϕ**_**9**_, and **ϕ**_**10**_ torsion angles describing the side chain, which is the flexible part of the bisabolol molecule. The **C1** and **H1’** atoms attached to the two stereogenic centers are highlighted in Figure 1 for easy visualization.

**Figure 1:**
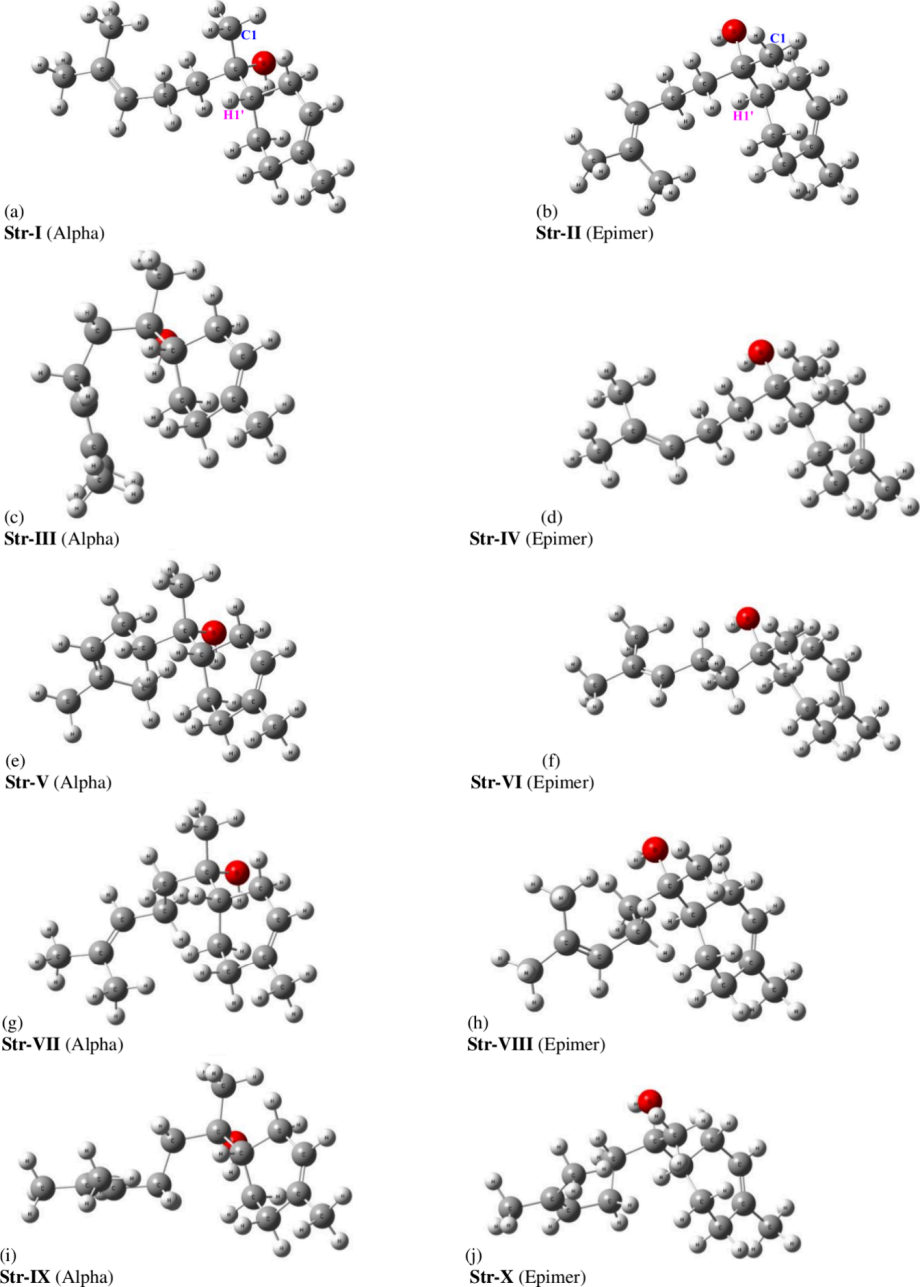
ωB97X-D/6–31G­(d,p)-PCM-Chloroform optimized structures for α-bisabol (**I**, **III**, **V**, **VII,** and **IX**) and its epimeric form (**II**, **IV**, **VI**, **VIII,** and **X**).

Table 2 reports relative energies (Δ*E*, Δ*H*, and Δ*G*), evaluated with respect to structure **I**, for all ten structures depicted in Figure 1. Geometry optimizations were carried out in the vacuum and included solvent effects using an implicit model (named PCM-Only) and adding two explicit solvent molecules close to the OH group, as expected (named PCM-2CHCl_3_). Thermodynamic properties were calculated for structures optimized in a vacuum, using the implicit solvation model (PCM-Only) and PCM-2CHCl_3_ optimized structures. The vacuum and PCM-nCHCl_3_ ΔE profiles may be better visualized in Figure 2. It can be observed that Δ*E* and Δ*H* values given in Table 2 are very similar indeed, and so we may take the ΔE calculated values approximately as enthalpy differences (Δ*H*).

**Table 2:** ωB97X-D/6-31G­(d,p)-PCM-nCHCl_3_ (*n* = 0, 2) and Vacuum Thermodynamic Data (**ΔE**, **ΔH**, **ΔG**/kcal mol^–1^) for α-Bisabol (Structures **I**, **III**, **V**, **VII,** and **IX**) and Its Epimeric Form (Structures **II**, **IV**, **VI**, **VIII,** and **X**)

	vacuum-OPT-Geom. (true minima)			PCM-only-OPT (true minima)			PCM-2CHCl_3_-OPT (true minima)		
strs.	Δ*E*	Δ*H*	Δ*G*	Δ*E*	Δ*H*	Δ*G*	Δ*E*	Δ*H*	Δ*G*
**I** (alpha)	0	0	0	0	0	0	0	0	0
**II** (epimer)	0.7	0.4	–0.9	0.0	0.1	0.5	–2.1	–1.6	1.1
**III** (alpha)	0.5	0.3	1.1	0.7	0.8	1.7	**-4.8**	**-4.5**	**-1.5**
**IV** (epimer)	0.5	–0.1	–0.9	0.8	0.6	–0.3	**-4.2**	**-3.5**	**-0.6**
**V** (alpha)	0.5	0.1	–0.5	0.6	0.8	0.8	–1.4	–1.0	2.0
**VI** (epimer)	0.2	–0.3	–0.9	0.1	0.2	0.2	–1.6	–0.6	2.6
**VII** (alpha)	2.4	2	1.5	2.5	2.8	3.1	4.7	5.0	5.0
**VIII** (epimer)	3.2	2.6	1.5	3.1	3.2	3.7	1.8	2.1	2.7
**IX** (alpha)	3.3	3	2.3	3.5	3.5	3.8	1.4	1.9	2.7
**X** (epimer)	2.5	2.1	1.6	2.3	2.5	3.2	0.7	1.1	2.9

**Figure 2:**
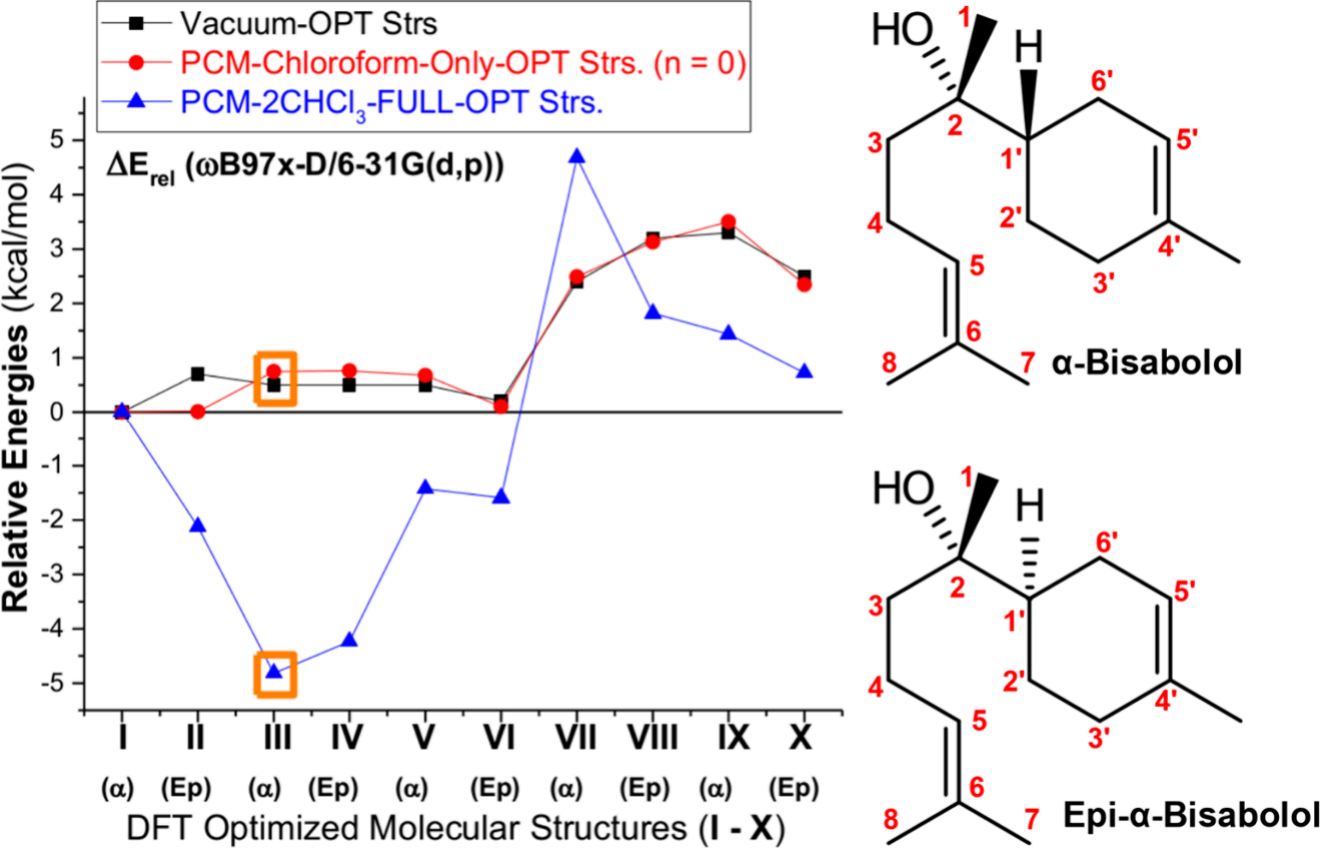
ωB97X-D/6–31G­(d,p)-PCM-nCHCl_3_ (*n* = 0, 2) and vacuum relative energies for ten distinct conformers of bisabolol.

It can be seen from Table 2 and Figure 2 that there is no noticeable difference between DFT-vacuum and DFT-PCM-Only ΔE and ΔH relative values with ΔG showing a sizable variation around 1.5 kcal mol^–1^. In both cases, structures **I** to **V** exhibited the lowest relative energies, while structures **VI** to **X** show larger values and may be considered in principle not favorable based on energetic grounds. ΔE and ΔH do not differentiate structures **I** to **VI**, only ΔG places structure **III** as less favorable. Adding two explicit solvent molecules (PCM-2CHCl_3_ relative energy values) substantially affected the energetic order. Now structure **III** (alpha) is the preferred one, followed by structures **IV** (epimer) and **VI** (epimer). This solvation model having two explicit CHCl_3_ solvent molecules close to the polar OH group, with the hydrogen atom of the CHCl_3_ molecule close to the OH group (HO···HCCl_3_) and the chlorine atom interacting with HO (OH···ClCHCl_2_), seems fine based on our chemical intuition (see Figure 4).

We can take structures **III** (Alpha) and **IV** (Epimer) as the relevant structures of bisabolol based on DFT-PCM-2CHCl_3_ relative energy results, among the ten distinct structures investigated here. For structures optimized in the vacuum and using the implicit solvent model (named PCM-Only), structures **I**, **II**, **III,** and **IV** can be considered degenerate since energy differences are below 1 kcal mol^–1^, which is within the estimated precision of DFT energies. The implicit solvation model does not distinguish between structures **I** to **IV**. The relative energy pattern shown in Figure 2 and Table 2 indicates that care is needed when using a Boltzmann distribution to average contributions of different conformations of the same molecule to calculate ^1^H NMR chemical shifts, a procedure frequently used, since energy values can vary significantly as a function of the solvent model used. This can be exemplified by the ωB97X-D/6-31G­(d,p) Boltzmann population for ten optimized structures located on the PES for α-bisabol and its epimeric form, using calculated **ΔE**, **ΔH**, and **ΔG** relative energy values (kcal mol^–1^), reported in Table 2. Boltzmann populations are significantly affected by explicit solvent effects in the calculation of relative energies. For DFT PCM-Only (*n* = 0) optimized structures, the largest Boltzmann populations (room temperature) evaluated with ΔE values from Table 2 are 27% (str. **I**), 26% (str. **II**), and 22% (str. **VI**), while for DFT -PCM-2-CHCl_3_ structures, the highest population is for structure **III:** 72%, followed by structure **IV** (26%). A similar population trend is obtained with ΔG values. Structure **III** has a Boltzmann population of only 7% at the DFT PCM-Only level.

RMSD values for ^1^H NMR chemical shifts with respect to experimental data (in CDCl_3_), evaluated at the B3LYP/6-31G­(d,p)- PCM-nCHCl_3_ level (*n* = 0, 2), are shown in Figure 3. RMSD results, including the OH proton (named all protons) and considering only CH_*n*_ protons (named CH_*n*_ protons), are reported in Figure 3a,b, respectively. We highlighted in Figure 3 structures **III** and **IV**, based on the relative energy values given in Table 2 and Figure 2, and structures having low RMSD values (**VII**, **VIII,** and **IX**). These can be considered relevant structures of bisabolol to exist in chloroform solution according to DFT calculations of the relative energy and ^1^H NMR chemical shifts. It can be seen from Figure 3 that RMSD patterns evaluated using implicit and explicit solvent models and including and excluding OH proton chemical shifts are very distinct. For example, structure **VII** is predicted as the most favored one at the PCM-Only (*n* = 0) level excluding OH protons, while structures **III**, **VIII,** and **IX** have roughly similar low RMSD values evaluated at the PCM-2CHCl_3_ level including OH protons. The deviation between DFT-PCM-2CHCl_3_ RMSD values for structures **III** and **IX** is only 0.03 ppm, certainly below the precision of DFT-based methods for NMR chemical shift calculations.

**Figure 3:**
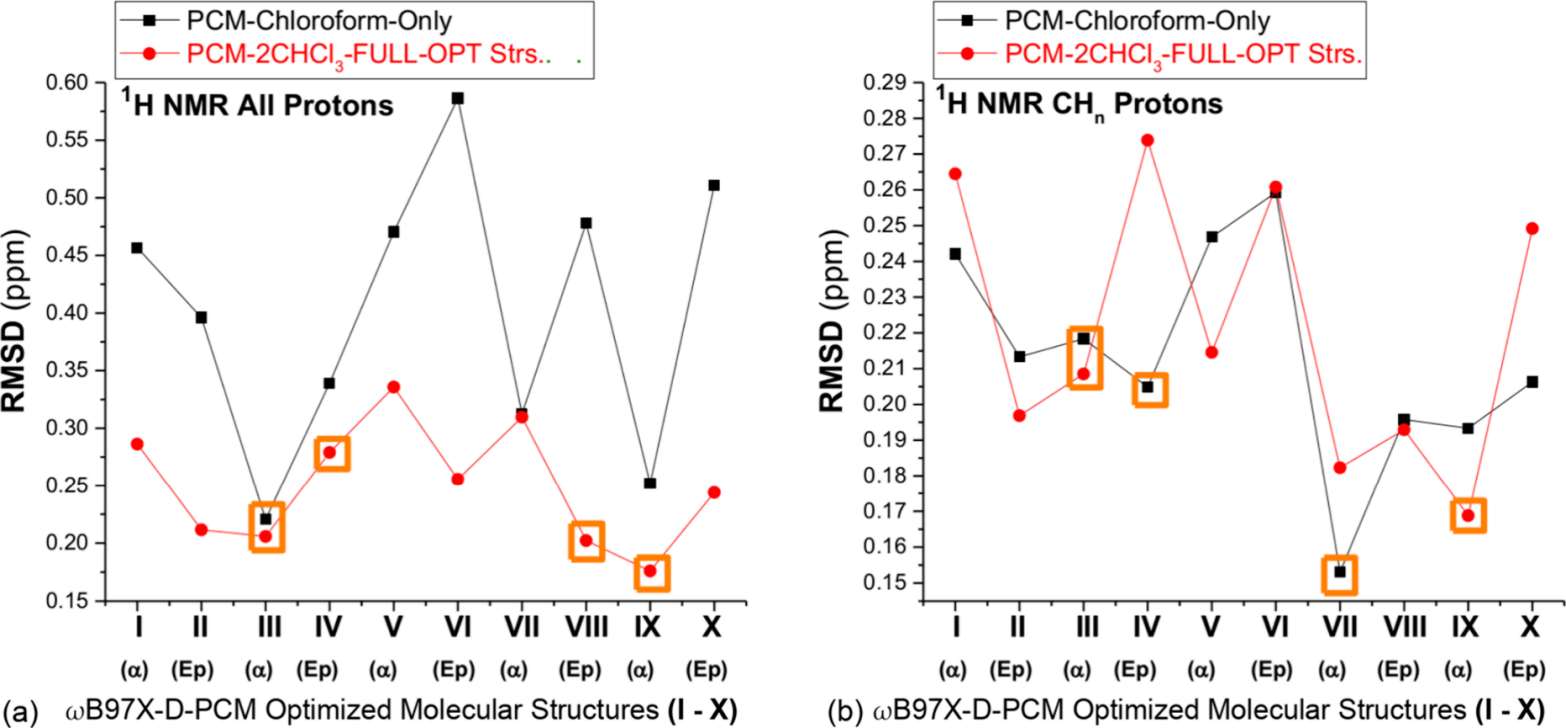
RMSD B3LYP/6-31G­(d,p)-PCM-*n*CHCl_3_ (*n* = 0, 1, 2) ^1^H NMR chemical shifts with respect to experimental data (in CDCl_3_). (a) All protons included (b) CH_*n*_ protons only.

ωB97X-D/6–31G­(d,p)-PCM-2CHCl_3_ optimized structures for five chosen conformers of bisabolol **III**, **IV**, **VII**, **VIII,** and **IX**), indicated in Figure 3, are shown in Figure 4. Intermolecular solute–solvent distances (Cl···H–O and C–H···O–H) are quoted. The C1 and H1’ atoms attached to the two stereogenic centers are highlighted, and the two configurations (alpha and epimer) can be visualized. This is a simple and adequate solvation model, based on our chemical intuition, to describe the explicit interaction of CHCl_3_ solvent molecules with the bisabolol solute.

**Figure 4:**
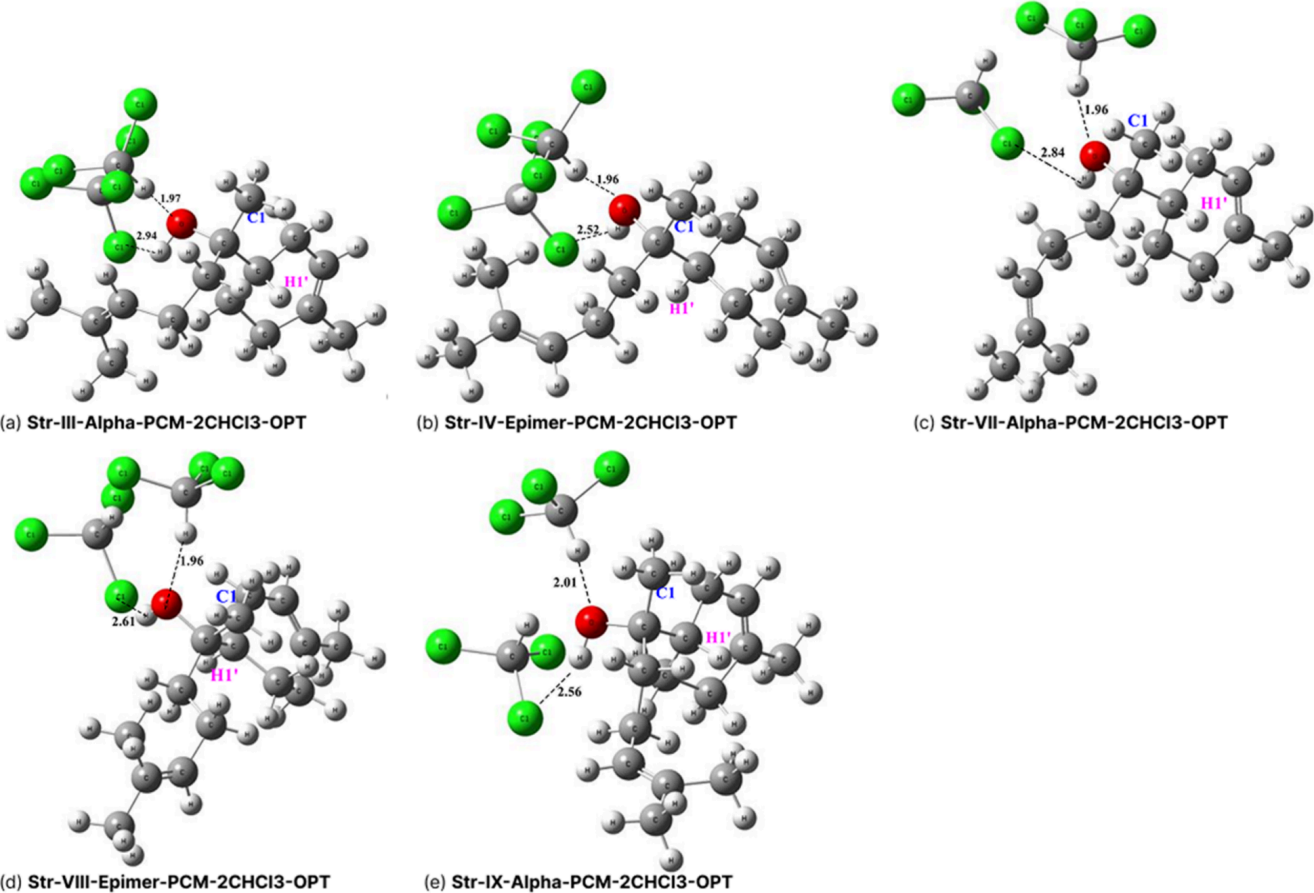
ωB97X-D/6–31G­(d,p)-PCM-2CHCl_3_ optimized main structures for α-bisabol (structures **III**, **VII,** and **IX**) and epimer-bisabol (structures **IV** and **VIII**).

The RMSD values reported in Figure 3 do not seem conclusive in the prediction of the preferred bisabolol structure in chloroform solution, and in our view, RMSD values calculated including OH protons should be recommended. Another way to use NMR chemical shift data for structural elucidation is through analysis of the experimental and theoretical ^1^H NMR profile. Therefore, B3LYP/6-31G­(d,p)-PCM-nCHCl_3_ (*n* = 0, 1, 2) ^1^H NMR spectra for selected structures **III, IV**, **VII**, **VIII,** and **IX,** shown in Figure 4, are reported in Figure S1 (Supporting Information), along with the experimental spectrum (in CDCl_3_) and RMSD values (in ppm). In all spectra presented in this article, only the centers of the experimentally obtained signals are represented, as the chemical shift value (the quantity we are seeking to reproduce here) reflects the chemical environment of the nucleus. We included DFT-PCM optimized structures having only one explicit CHCl_3_ solvent molecule for reasons of comparison. The remarkable effect of the explicit solvent on the ^1^H NMR chemical shifts for the OH proton can be clearly seen. Using the implicit solvent model (named PCM-OPT) leads to a significant underestimation of the OH chemical shift value for all structures compared to the experimental value (1.53 ppm). Only for structure **III,** a small deviation between calculated and experimental (1.28 ppm) OH chemical shift value is predicted, the NMR profile for structure **III** being relatively closer to the experimental pattern among all ten DFT-PCM-Only structures. The ^1^H NMR spectra for structure **III** (PCM-*n*CHCl_3_; *n* = 0, 1, and 2) are shown in Figure 5a,c.

**Figure 5:**
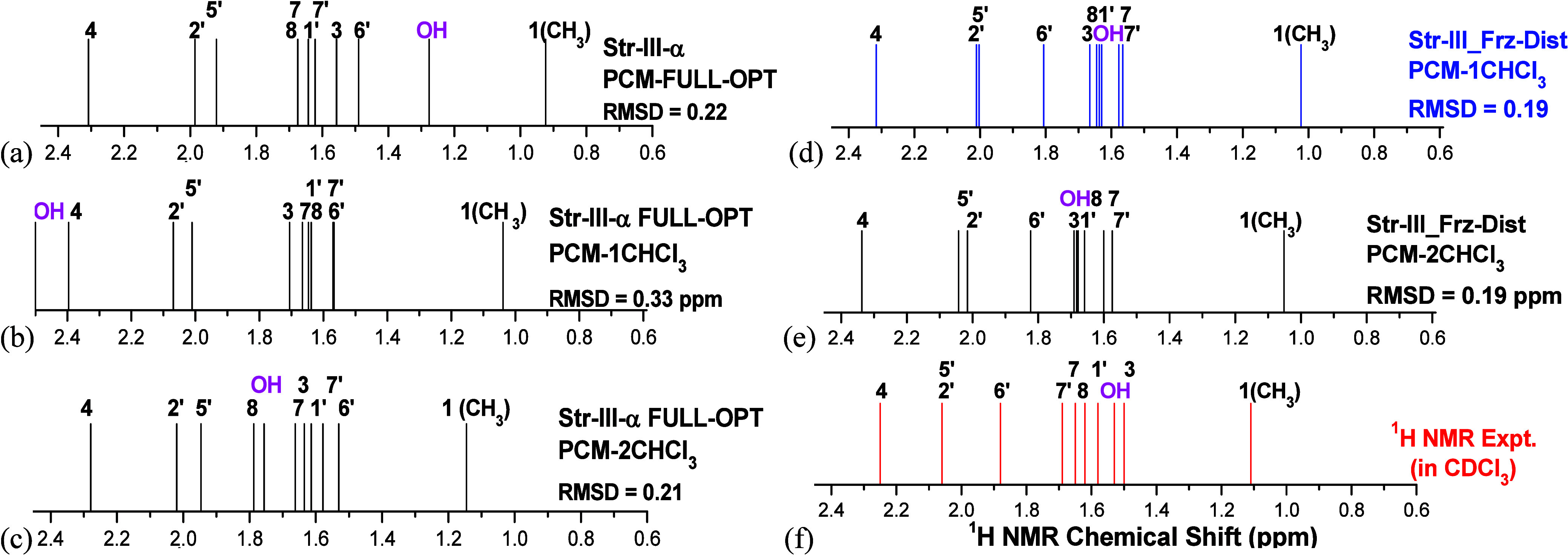
(a–c) B3LYP/6-31G­(d,p)-PCM-nCHCl_3_ (*n* = 0, 1, 2) for fully optimized structures and ^1^H NMR spectra for structure **III**. (d, e) B3LYP/6–31G­(d,p)-PCM-nCHCl_3_–Frz-Dist (*n* = 1, 2) ^1^H NMR spectra for structures **III**. (f) Experimental spectrum.

The addition of only one explicit solvent CHCl_3_ molecule in the input for geometry optimization resulted in a very large increase in the OH chemical shifts for structure **III**, a moderate increase for structures **IV** and **IX**, and practically no change for structures **VII** and **VIII**. The best overall agreement with the experimental spectrum is obtained for optimized structure **III** having two explicit solvent molecules (Figure S1c), but the positions of protons 6’, 8, and OH are swapped. However, the OH chemical shift for PCM-2CHCl_3_ structure **III** (1.76 ppm) is much closer to the experimental value (1.53 ppm) than the respective PCM-1CHCl_3_ structure (2.58 ppm), which is significantly overestimated. These results show that there is an interesting balance between the DFT-PCM calculated OH chemical shift value and the specific position of the CHCl_3_ molecule relative to the solute.

As already mentioned, structure **III** shows the best overall agreement with the experimental ^1^H NMR spectrum among the ten DFT-PCM optimized bisabolol structures. We employed a frozen-geometry approach, designated “Frz-Dist”, to obtain a better match between the experimental and theoretical ^1^H NMR data. This procedure consists of manually placing a CHCl_3_ molecule in the proximity of the OH group without subsequent geometry optimization. Through a trial-and-error procedure (artisanal), we found an adequate position of the CHCl_3_ solvent molecule around the OH group for structure **III** that reproduces correctly the experimental ^1^H NMR spectrum for all protons. To reach an almost perfect agreement with the experiment, an additional manual rotation of the OH group by 25° from the optimized torsion angle was made (see Figure S2a, Supporting Information, highlighted in blue color). This structure also has a low RMSD value (0.19 ppm). This same CHCl_3_ frozen solvent geometry around the solute obtained successfully for structure **III** was used for the NMR calculation of structures **IV**, **VII**, **VIII,** and **IX** with no improvement in the agreement with the experiment observed. The same procedure was repeated for PCM-2CHCl_3_ structures, and again structure **III** exhibited a very good agreement with the experimental spectrum. These final DFT-PCM-nCHCl_3_ NMR spectra are shown in Figure S2.

Looking at Figure S2c, it can be seen that the 2CHCl_3_–Frz-Dist spectrum is just a refinement of the PCM-2CHCl_3_ fully optimized structure spectrum of structure **III** (Figure S1e), which improves the OH and CH_*n*_ NMR signals. The match between the theoretical spectrum and experimental NMR profile is almost perfect (RMSD < 0.20 ppm). The other PCM-2CHCl_3_–Frz-Dist calculated spectra (structures **IV**, **VII**, **VIII,** and **IX**) exhibited large deviations from the experimental ^1^H NMR profile (although some of them show a small RMSD value) and therefore can be safely disregarded as the predominant bisabolol structure to be present in chloroform solution. The fact that the RMSD trend is not always in line with the ^1^H NMR profile agreement with experiment is an interesting result, raising a question about the reliability of a criterion used to determine the preferred molecular structure to be present in solution through analysis of NMR data. The ^1^H NMR spectra for structure **III** (PCM-1CHCl_3_ and PCM-2CHCl_3_) are shown in Figure 5d,e, and the experimental profile is shown in Figure 5f.

*m*PW1PW91/6-31G­(d) spectra, for vacuum optimized Alpha and Epimer evaluated with Boltzmann averaged structures from ref 7 are also shown in Figure S2j,l, respectively (OH protons were excluded), where the better agreement with the experimental ^1^H NMR profile for the Alpha structure (equivalent to our structure **III**, highlighted in pink color) can be visualized. Although the RMSD value of 0.13 ppm is smaller than our value of 0.17 ppm (OH protons included) for structure **III**-PCM-1CHCl_3_–Frz-Dist, the positions of protons H6’ and H3 are incorrectly predicted by the Boltzmann averaged spectrum from ref 7. The lowest RMSD value may not always agree with the correct ^1^H NMR pattern prediction. Our PCM-1CHCl_3_–Frz-Dist spectrum for structure **III** nicely predicted the position of the NMR signal of all protons, providing strong evidence of the existence of the Alpha stereoisomer in chloroform solution.

Our theoretical approach is based on quantum chemical calculations at a molecular level within the Born–Oppenheimer approximation, not a statistical method as used in ref 7, and so the predominant molecular structure in solution can be predicted. We showed that a direct comparison between the theoretical (PCM-nCHCl_3_) and experimental (in CDCl_3_ solution) ^1^H NMR profile can be more suitable for structural elucidation than analysis of statistical indices.

The deviation from the experimental NMR data can also be analyzed through a fitting line procedure. We selected DFT-PCM-nCHCl_3_^1^H NMR data for the two main structures **III** and **IV** (Figure 2) to analyze deviation from experimental data. The results are shown in Figure 6, which strongly corroborates our predictions based on the analysis of the ^1^H NMR profiles.

**Figure 6:**
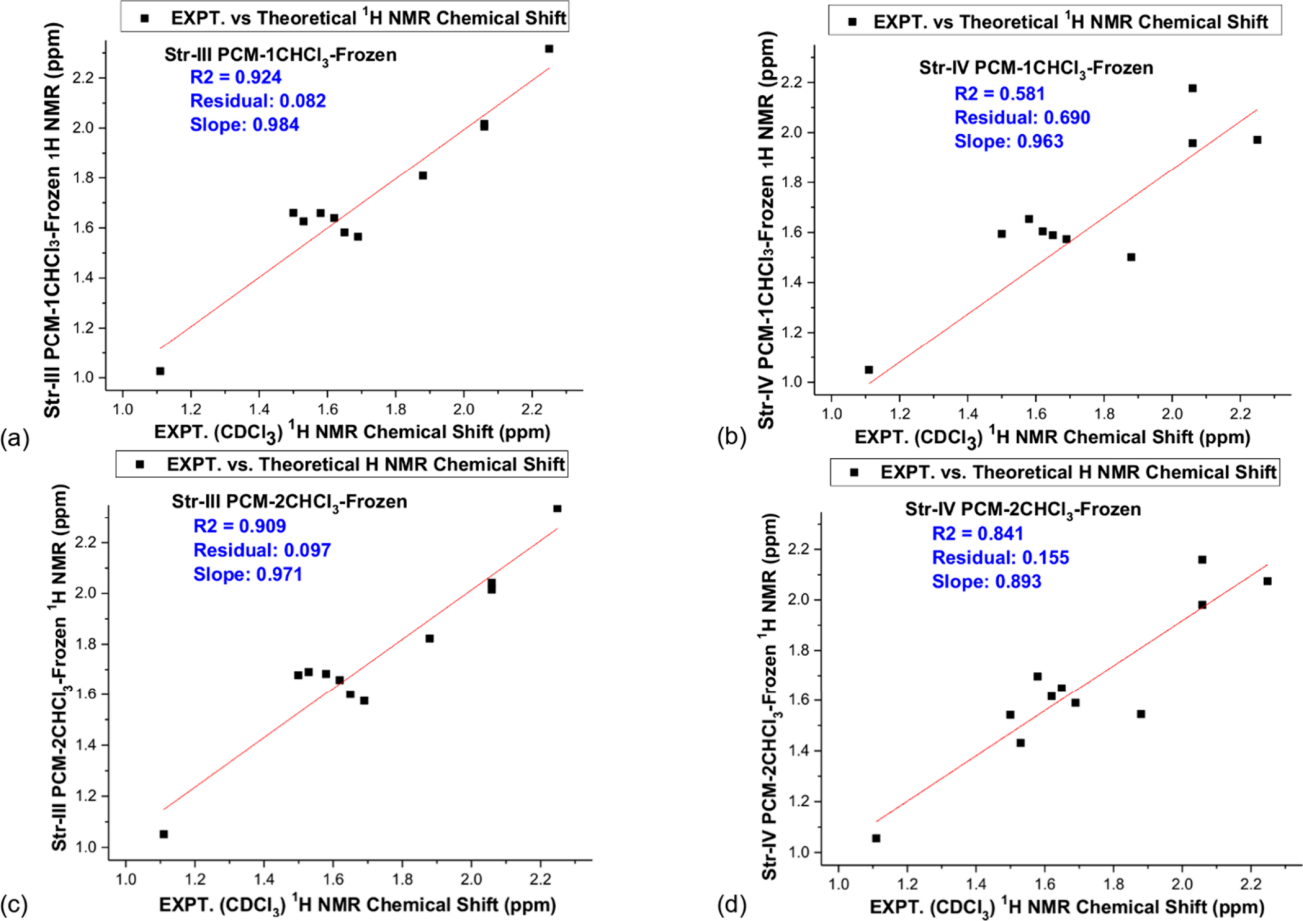
Theoretical (DFT/PCM-nCHCl_3_) vs Experimental (CDCl_3_) ^1^H NMR chemical shift linear fitting. (a) **Str-III**-PCM-1CHCl_3_ (RMSD = 0.19) (b) **Str-IV**-PCM-1CHCl_3_ (RMSD = 0.20) (c) **Str-III-**PCM-2CHCl_3_ (RMSD = 0.19) (d) **Str-IV**-PCM-2CHCl_3_ (RMSD = 0.18).

It is opportune to mention that the use of NMR chemical shifts for structural elucidation of organic compounds, particularly stereochemical assignments for diastereomers, was reviewed in detail four years ago.^17^ The use of DFT calculations and statistical methods (and an ANN) to assist experimental NMR data, aiming at the identification of diastereomeric structures, was properly discussed. The combination of advanced probabilistic methods, such as CP3 and DP4, with computational NMR chemical shifts for structure validation has been discussed, along with the progress achieved in structural identification of diastereomeric species based on the analysis of theoretical NMR properties. The development of the DP4+ and ANN PRA methods was highlighted. It was concluded in ref 17 that in some cases, only the synergy of synthesis, standard/anisotropic NMR and chiroptical measurements, DFT/GIAO-based calculations, and advanced statistical analysis of available NMR data can reveal the correct stereostructure of complex diastereomeric systems, which is also true for elucidating the relative/absolute configuration of other bioactive compounds.

The DP4+ method has been shown to be very useful in the correct elucidation of structures of natural organic compounds, working better than the usual analysis of statistical indices, despite using the same input, i.e., deviations between theoretical and experimental chemical shift values. The DP4+/ANN-PRA tools are statistical in nature, and our approach is based on quantum chemical calculations of NMR chemical shifts for DFT-PCM-nCHCl_3_ optimized structures located on the Born–Oppenheimer PES. Instead of analyzing statistical indices, which measure discrepancies between theoretical and experimental chemical shift values, we analyze the agreement between theoretical and experimental (in CDCl_3_) ^1^H NMR spectra, which is not a statistical approach and seems very sound to us, allowing a direct comparison with experimental observations in solution. We think this can be seen as a demonstration of the robustness of our approach. The experimental ^1^H NMR profile is a key information, and finding the correct molecular structure that reproduces the experimental spectrum is not a trivial procedure of just optimizing the geometry of a random solvated input structure obtained, for example, from a classical simulation method. In previous work,^7^ the aim of using the DP4+/ANN-PRA tools was to determine the relative configuration of the natural product alpha bisabol, which was successfully done. Our approach allows us a successful prediction of the predominant molecular structure present in solution. The good agreement between the DP4+/ANN-PRA and our predictions of the relative configuration of alpha bisabol is a good result, showing the pertinence of both distinct approaches.

At this point, it seems appropriate to evaluate the effect of the choice of DFT functional to calculate relative energies and ^1^H NMR chemical shifts of different conformers (or isomers) of the same molecule. PCM-Only and PCM-1CHCl_3_ single-point relative energy values using ωB97X-D,^12^ B3LYP,^15,16^ *m*PW1PW91 ^18^ and M06-2X^19^ functionals are reported in Table S1 (Supporting Information) for the main structures **III** and **IV**. While for structures **I**, **II,** and **IV**, relative energies do not deviate significantly for the four DFT functionals, large discrepancies are observed for structure **III**, regarding B3LYP and *m*PW1PW91 values, which predicted this structure to be quite unfavorable, with B3LYP and *m*PW1PW91 showing similar predictions. On the other hand, there is an overall agreement between ωB97X-D and M06-2X functionals as far as the relative energy trend is concerned. The B3LYP and *m*PW1PW91 functionals show a somehow surprisingly very large deviation from the ωB97X-D and M06–2X functionals, both with respect to the size and direction of relative energies. These results indicate that the B3LYP and *m*PW1PW91 functionals may not be very adequate to evaluate energy differences between two conformers of the same molecule, and so Boltzmann population calculated with the *m*PW1PW91 functional, as has been done in the previous article of bisabolol by Dos Santos Jr. et al.^7^ may lead to questionable results. This behavior of the B3LYP functional to underestimate relative energies was shown some years ago regarding the prediction of binding energies for inclusion complexes.^20^ The B3LYP and *m*PW1PW91 relative energy results for structure **III**, the only case where the disagreement with the overall trend predicted by other functionals is evident, show how sensitive the calculation energy values can be to the chosen DFT functional.

A comparative theoretical PCM-1CHCl_3_^1^H NMR spectra for α-bisabol (a randomly chosen structure **III**) and its epimeric form (structure **IV**), calculated using distinct DFT functionals (B3LYP, ωB97X-D, *m*PW1PW91, and M0602x) and the experimental spectrum (in CDCl_3_), is shown in Figure S3 (Supporting Information). The ^1^H NMR profiles for the B3LYP, ωB97X-D, and *m*PW1PW91 functionals are very similar, there is only a translation of the whole spectrum, but the M06–2X functional shows a large deviation in the NMR profile, indicating that it does not seem adequate to calculate NMR chemical shifts. In general, as observed previously for nitrogenated compounds^21^ changing the DFT functional does not affect substantially the ^1^H NMR profile with roughly the same trend in the NMR signals being predicted.

Lastly, the solvent effects on the molecular structure can be assessed through a comparison of selected torsion angles (°) calculated for the chosen structures **III** and **IV**. Results for structures optimized at the DFT-PCM-*n*CHCl_3_ (*n* = 0, 1, 2) level are given in Table S2. It can be seen that no drastic changes are observed in the sense that the solute molecular structure is reasonably preserved in the presence of explicit solvent molecules. Only one specific torsion angle **ϕ**_**10**_ [C6.C5.C4.C3] shows very large variations due to solute–solvent interactions. This relates to the flexible side carbon chain, which is more exposed to interaction with solvent molecules.

Finally, we make some final remarks regarding solvent effects on DFT calculations of ^1^H NMR chemical shifts. Our results allowed us to address interesting points and reach some conclusions. The standard procedure in quantum chemical (mostly at the DFT level) studies is comparison with experimental data, which are usually reported in solution. Therefore, solvent effects must be included in DFT calculations, which can be done using implicit solvent (continuum models) or by including explicit solvent molecules in the geometry optimization procedure (the latter should be closer to the experimental conditions). Our experience in various other theoretical studies with large organic molecules showed that finding an adequate position of explicit solvent molecules around the solute is not a trivial computational task, which is corroborated by the results reported in the present work. Our main findings are summarized in Figure 7 summarizes the main findings for structure **III** of the bisabolol molecule, which exemplifies very well the relevance of using the experimental ^1^H NMR profile to elucidate the preferred molecular structure in chloroform solution and how the specific solvation model used affects the agreement with experimental data. We believe that the most probable molecular structure present in solution should reproduce faithfully the experimental NMR profile, and to reach an almost perfect agreement with the experimental ^1^H NMR pattern, we used a trial-and-error (artisanal) procedure, varying manually the position of the CHCl_3_ molecule around the solute until a satisfactory agreement with the experiment was found. Then, a refinement in the spatial orientation of the solvent molecule was done, and the final solvated structure (with the frozen solvent geometry) was obtained. The best DFT-PCM-1CHCl_3_^1^H NMR spectrum and molecular structure are shown in Figure 7a. A similar procedure was repeated for the DFT-PCM 2CHCl_3_ structure, and the same very good agreement with the experimental spectrum was predicted (Figure 7b). We then fully reoptimized the two frozen solvent structures (Frz-Dist) and calculated the NMR spectrum. The results are shown in Figure 7c,d, where it can be seen that the nice agreement with experiment is totally broken when the geometry is fully optimized, relaxing the solvent geometry, the 8e effect being more drastic for the PCM-2CHCl_3_ structure. The results shown in Figure 7 strongly indicate that standard geometry optimization of an arbitrary input structure containing explicit solvent molecules does not often lead to the correct optimized solvated structures that reproduce precisely the experimental ^1^H NMR profile. An artisanal procedure to place solvent molecules around the solute, as used in this work, seems adequate, although it may not be easily reproducible. A true minimum energy structure located on the PES for explicitly solvated structures may not correspond to the molecular structure present in solution, which leads to a good match between theoretical and experimental NMR spectra. This is corroborated by a comparison between the DFT ^1^H NMR spectrum for structure **III** (PCM-1CHCl_3_–Frozen-Dist), exhibiting the best agreement with experiment, and the corresponding fully optimized true minimum energy structure (PCM-1CHCl_3_–Frozen-Dist-FULL-OPT) shown in Figure S4 (Supporting Information). What our result tells us is that the agreement with the experimental NMR spectra is a very strong indication that the explicit solvent molecules are in the right position around the solute.

**Figure 7:**
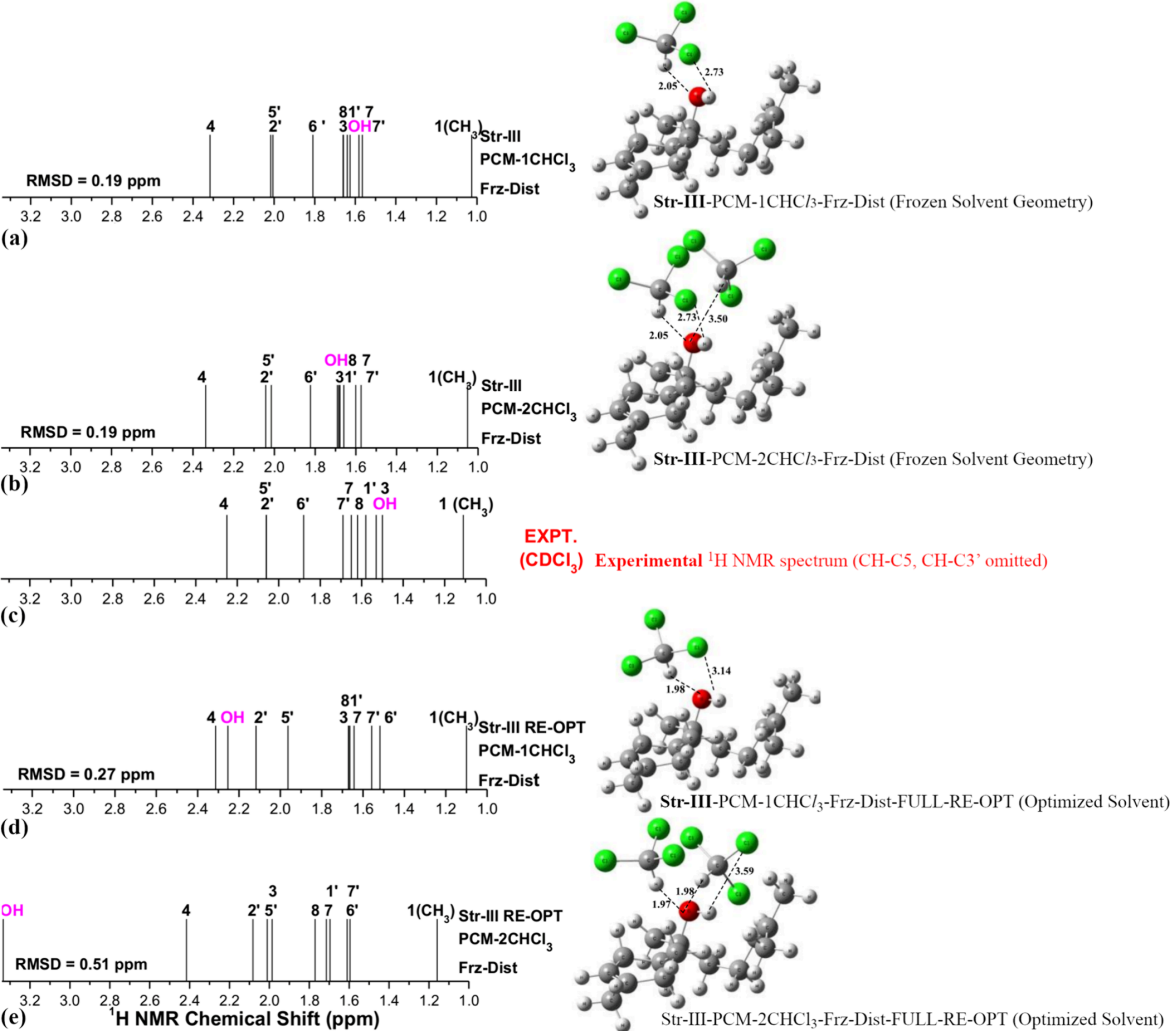
B3LYP/6-31G­(d,p)-PCM-nCHCl_3_-(*n* = 1, 2) ^1^H NMR spectra for bisabolol structure **III**, calculated using two distinct geometries: (a, b) PCM-nCHCl_3_–Frz-Dist (the position of solvent molecules around the solute were not optimized, but kept frozen at fixed spatial orientation and (d, e) the PCM-nCHCl_3_–Frz-Dist structures from (a, b) were now fully optimized (named RE-OPT). Experimental ^1^H NMR spectrum (in CDCl_3_) is shown in (c).

The artisanal mode of placing solvent molecules around a solute has been addressed in a previous work on the molecular structure of the antibiotic AZM.^22^ In the artisanal procedure of placing explicit solvent molecules (CHCl_3_) around the AZM solute in geometry optimizations, we selected initial positions of CHCl_3_ molecules around C–OH protons using our chemical intuition. To confirm that our assumption was reasonable, we performed molecular dynamics (MD) simulation using chloroform as the solvent. Analysis of MD results reveals that the most probable spatial orientation of CHCl_3_ molecules around the AZM solute was in full agreement with our DFT-PCM-5CHCl_3_ optimized geometries (see Figure S5, ref 22, therefore validating our approach.

## Conclusions

The assignment of the relative configuration of natural products, particularly when stereogenic centers are present, as is the case for αBis (alpha and epimeric form), can be a difficult experimental task. Recently, a theoretical NMR study was reported,^7^ making use of the DP4+ and ANN-PRA new tools, which are based respectively on Bayesian probability and ANNs, to handle NMR chemical shift analysis, using DFT methodology to generate many optimized structures (in the vacuum) of the α and epimer stereoisomers, followed by Boltzmann averaged NMR chemical shift calculations as input by the DP4+ and ANN-PRA computational algorithms. The alpha form of bisabolol was predicted as preferred in chloroform solution, but no information on the spatial arrangement of the molecular structure present in the NMR experiment (in CDCl_3_) could be obtained. In our view, there is a strong dependence on the NMR input data, as happens with any computational procedure with a statistical nature

In this work, we addressed the assignment of the relative configuration of αBis through standard DFT calculations of molecular structure and NMR chemical shifts in chloroform solution using the implicit PCM solvent model, with the inclusion of explicit chloroform solvent molecules named PCM-nCHCl_3_ (n = 0, 1, 2). Analysis of the results obtained with four chosen DFT functionals (ωB97X-D, B3LYP, *m*PW1PW91, and M06–2X) was done to assess the influence on calculated relative energies and NMR chemical shifts. Based on our results, B3LYP and *m*PW1PW91 functionals may not be recommended to evaluate relative energies of conformers of the same molecule, while M06–2X is not appropriate for NMR calculations. A search for true minimum energy structures on the PES for bisabolol was carried out through a scan procedure varying relevant torsion angles with ten distinct minima being found on the PES (five alpha and five epimeric forms) using the ωB97X-D/6–31G­(d,p)-PCM-Chloroform approach. We believe that these ten structures may be considered representative of the relevant bisabolol conformers in solution. A comparative analysis of the theoretical and experimental (CDCl_3_) ^1^H NMR profile was used as a strategy to elucidate the predominant molecular structure in chloroform solution, which should faithfully reproduce the experimental ^1^H NMR pattern. This is a procedure rather different from the approach used before for bilabolol,^7^ but it corroborated the previous analysis performed with the DP4+ and ANN-PRA tools in the sense that the same preferred stereoisomer form (alpha) was predicted.

The novelty in our approach is that we are able to determine the preferred molecular structure of the predominant alpha stereoisomer of bisabolol in chloroform solution, which very likely is the one that will interact with biological targets, besides finding the relative configuration of αBis, This could not be done in the previous work^7^ where Boltzmann average NMR chemical shifts evaluated for a large number of candidate bisabolol structures leading to the best agreement with experimental data were reported (but not a molecular structure) and used as a criterion for the exclusion of the epimer stereoisomer form. In this sense, the present work complements the work reported on ref 7.

Some points deserve our attention regarding the solvent effects. First, relative energies evaluated with the implicit solvent model (PCM-Only) are like DFT results evaluated in the vacuum, with no substantial changes being observed. As reported recently,^21^ the inclusion of explicit solvent molecules is required for the correct prediction of ^1^H NMR chemical shifts for N–H groups using DFT-PCM calculation, and the same holds for O–H protons. In the case of bisabolol, the experimental NMR signal for the O–H protons (1.53 ppm) can be used to help identify the predominant molecular structure present in solution. Evaluation of RMSD values, including OH protons, brings more information than CH_n_ protons only. Using two explicit CHCl_3_ solvent molecules in the DFT-PCM geometry optimization procedure leads to a reasonable agreement with the experimental ^1^H NMR profile. In addition, the explicit solvent effect on the solute molecular structures is very small, and essentially the same conformation is obtained when the geometry is optimized at the PCM-Only or PCM-nCHCl_3_ level of calculation. We used an artisanal trial-and-error procedure to select the ideal position of one CHCl_3_ molecule around the OH group for structure **III** to improve agreement with the experimental NMR pattern, and an almost perfect match was obtained. Using this same frozen geometry of the CHCl_3_ solvent molecule (named Frz-Dist) for the other ten plausible molecular structures allowed us to assess the solvent effect on the ^1^H NMR spectrum in a consistent way. Through this procedure, we unambiguously determined that the alpha structure **III** of bisabolol is predominant in chloroform solution and should be used in further computational simulation of interactions in biological media utilizing quantum chemical methods.

## Supplementary Material



## Notes

Additional figures and tables, as well as the experimental section, and optimized Cartesian coordinates of all structures (PDF)
