# Supplementary material for: Determination of the Preferred Stereoisomer of Natural Product Bisabolol in Chloroform Solution through Quantum Chemical Calculations of 1H NMR Chemical Shifts
Source: ACS Omega. 2025 Oct 13;10(42):50445–56. doi: 10.1021/acsomega.5c07923 (PMC12573000; doi:10.1021/acsomega.5c07923)
Supplement: Supplementary file 1 [file ao5c07923_si_001.pdf]

SUPPLEMENTARY MATERIAL

DETERMINATION OF THE PREFERRED STEREOISOMER OF NATURAL PRODUCT BISABOLOL IN CHLROFORM SOLUTION THROUGH QUANTUM CHEMICAL CALCULATIONS OF ^{1}H NMR CHEMICAL SHIFTS

Haroldo C. Da Silva[a,b,c], Lucas H. Martorano[d], Fernando M. Dos Santos Jr.[d], Wagner B. De Almeida[a,b,*]

[a] Programa de Pós-Graduação em Química (PPGQ), Instituto de Química, Universidade do Estado do Rio de Janeiro (UERJ), Maracanã, Rio de Janeiro, RJ, Brazil, 20550-013

[b] Laboratório de Química Computacional e Modelagem Molecular (LQC-MM), Departamento de Química Inorgânica, Instituto de Química, Universidade Federal Fluminense (UFF), Outeiro de São João Batista s/n, Campus do Valonguinho, 24020-141, Centro, Niterói, RJ, Brazil. 24040-141

[c] Laboratório de Química Teórica e Simulação Molecular (LQTSM), Departamento de Físico-Química, Instituto de Química, Pavilhão Haroldo Lisboa da Cunha, Universidade do Estado do Rio de Janeiro (UERJ), Rua São Francisco Xavier, 524, 20550-013, Maracanã, Rio de Janeiro, RJ, Brazil.

[d] Departamento de Química Orgânica, Instituto de Química, Universidade Federal Fluminense (UFF), Outeiro de São João Batista s/n, Campus do Valonguinho, 24020-141, Centro, Niterói, RJ, Brazil. 24040-141

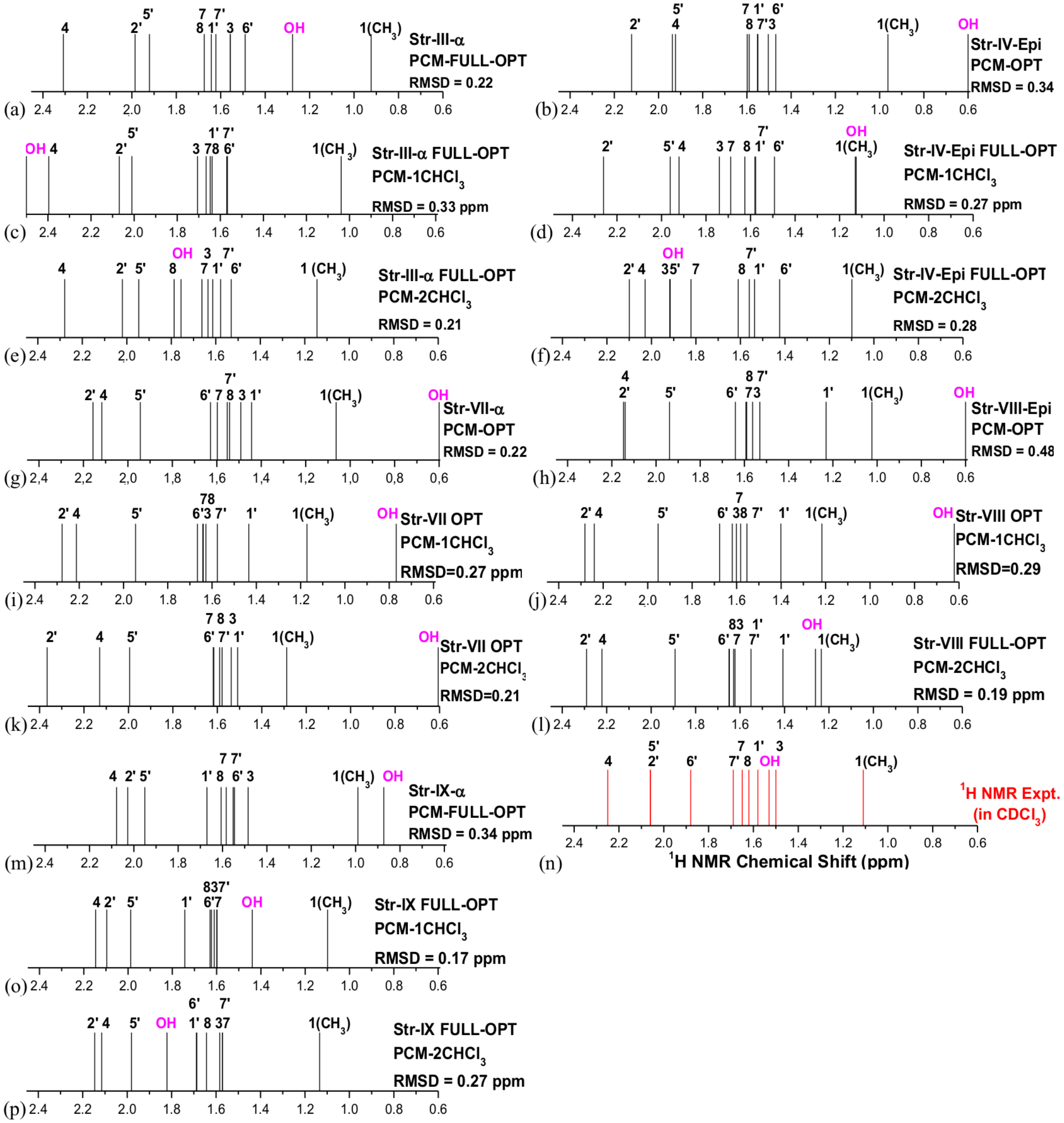

Figure S1. B3LYP/6-31G(d,p)-PCM-n$CHCl_3$ (n = 0, 1, 2) for fully optimized structures ^1H-NMR spectra (a-o). Experimental spectrum (n).

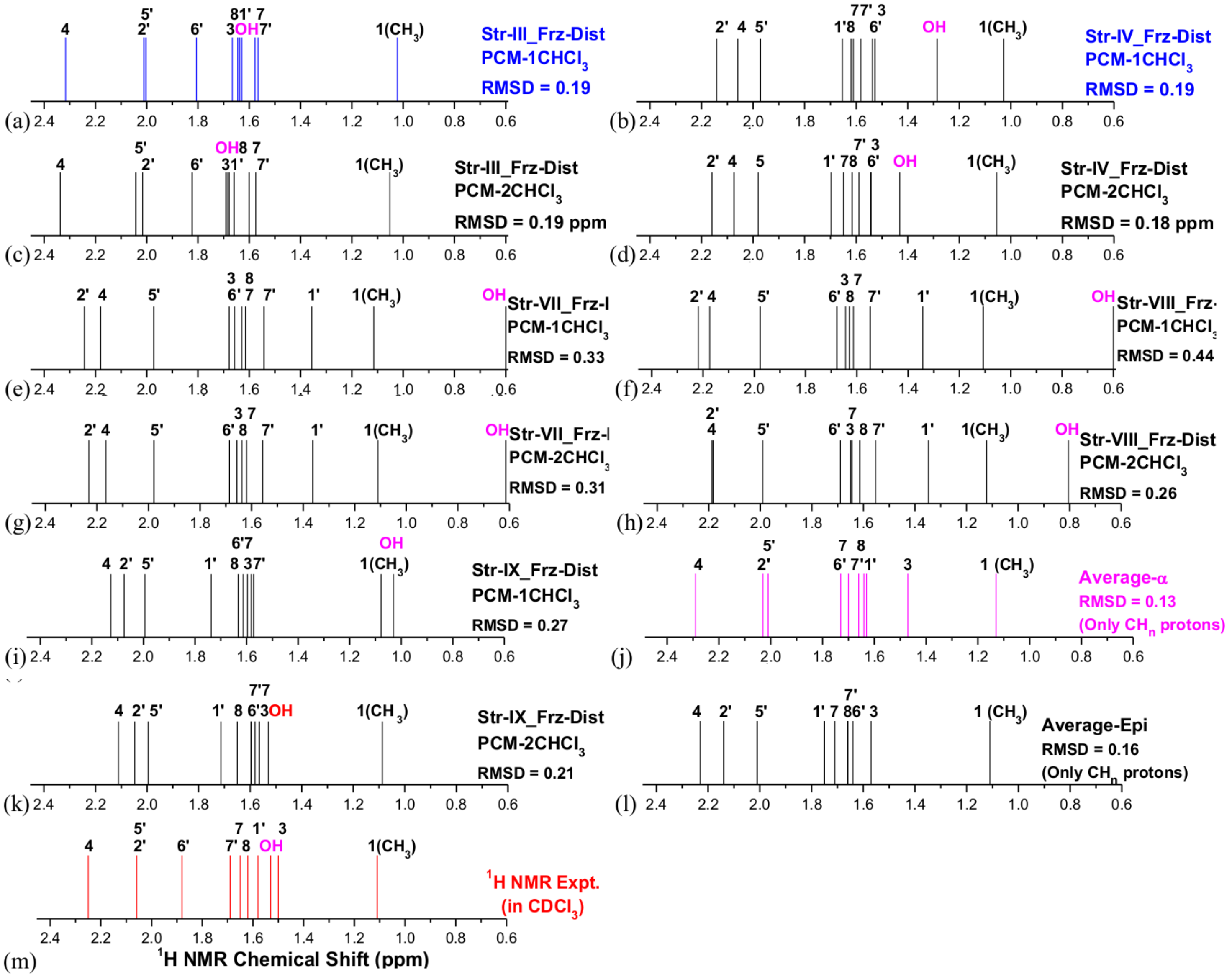

Figure S2. B3LYP/6-31G(d,p)-PCM-nCHCl3-Frz-Dist (n = 1, 2) ^{1}H-NMR spectra for structures **III**, **IV, VII**, **VIII** and **IX.** Spectra for Boltzmann average structures from ref.[7] (j-l). Experimental spectrum (in CDCl3) is also shown (m).

Table S1. ωB97x-D/6-31G(d,p)-PCM-Chloroform relative thermodynamic data (**ΔE, ΔH, ΔG** / kcal mol^{-1}) for α-bisabol (**Str-I**, **Str-II**, **Str-V** , **Str-VI** and **Str-VII**) and its epimer form (**Str-III**, **Str-IV**, **Str VIII**, **Str-IX** and **Str-X**) .

	**PCM-Only-OPT**				**PCM-OPT-1CHCl3-Frozen**			
	ωB97x-D	M06-2x[a]	B3LYP[b]	MPW1PW91[c]	ωB97x-D	M06-2x[a]	B3LYP[b]	MPW1PW91[c]
**I** (Alpha)	0.0	0.0	0.0	0.0	0.0	0.0	0.0	0.0
**II** (Epimer)	0.0	0.4	-0.1	0.1	-1.2	-0.6	-0.8	-0.7
**III** (Alpha)	0.7	0.7	**2.3**	**2.0**	-2.7	-1.8	**1.9**	**1.3**
**IV** (Epimer)	0.8	1.0	-0.5	-0.3	-1.5	-1.4	-1.9	-1.7

[a] M06-2x-PCM//ωB97x-D/6-31G(d,p)-PCM relative energy values (double slash means a single-point calculation).
[b] B3LYP/6-31G(d,p)-PCM//ωB97x-D/6-31G(d,p)-PCM relative energy values (double slash means a single-point calculation)
[c] MPW1PW91-PCM//ωB97x-D/6-31G(d,p)-PCM relative energy values (double slash means a single-point calculation).

Table S2. ωB97x-D/6-31G(d,p)-PCM-nCHCl$_3$ (n = 0, 1, 2) optimized selected torsion angles (ϕ$_i$ / °) for α-bisabol (**Str-III**) and its epimer form (**Str-IV**)

				Stereogenic		Lateral Chain					OH
**Structures**	ϕ_1	ϕ_2	ϕ_3	ϕ_4	ϕ_5	ϕ_6	ϕ_7	ϕ_8	ϕ_9	ϕ_{10}	ϕ_{11}
**III-PCM**	-149.9	-172.1	15.3	**69.4**	-30.4	85.8	-149.9	-58.4	-64.6	138.8	69.3
**III-PCM-1CHCl$_3$**	-155.4	-171.2	14.5	**70.9**	-35.7	80.4	-155.4	-64.0	-60.5	134.7	74.4
**III-PCM-2CHCl$_3$**	-151.7	-173.8	14.3	**68.3**	-31.2	85.1	-151.7	-66.7	-64.7	134.3	73.1
**Structures**	ϕ_1	ϕ_2	ϕ_3	ϕ_4	ϕ_5	ϕ_6	ϕ_7	ϕ_8	ϕ_9	ϕ_{10}	ϕ_{11}
**IV-PCM**	175.4	174.7	-14.8	**-69.1**	-61.5	54.7	175.4	-52.9	-173.0	102.3	51.1
**IV-PCM-1CHCl$_3$**	176.3	176.6	-12.9	**-66.6**	-59.6	56.8	176.3	-51.6	-160.7	76.5	42.0
**IV-PCM-2CHCl$_3$**	175.0	177.0	-12.8	**-66.6**	-60.5	55.6	175.0	-51.0	-155.2	77.0	43.3

ϕ_1 : [C2'.C1'.C2.C3]; ϕ_2 : [C3'.C2'.C1'.C2]; ϕ_3 : [C6'.C5'.C4'.C3']; **ϕ_4 : [H,C1'.C2'.C3']; ϕ_5 : [C1.C2.C1'.C2'];**
ϕ_6 : **[O.C2.C1'.C2']** ; ϕ_7 : [C3.C2.C1'.C2'] ; ϕ_8 : [C4.C3.C2.C1']; ϕ_9 : [C5.C4.C3.C2]; ϕ_{10} : [C6.C5.C4.C3]; ϕ_{11} ; : [H,O,C2.C1']

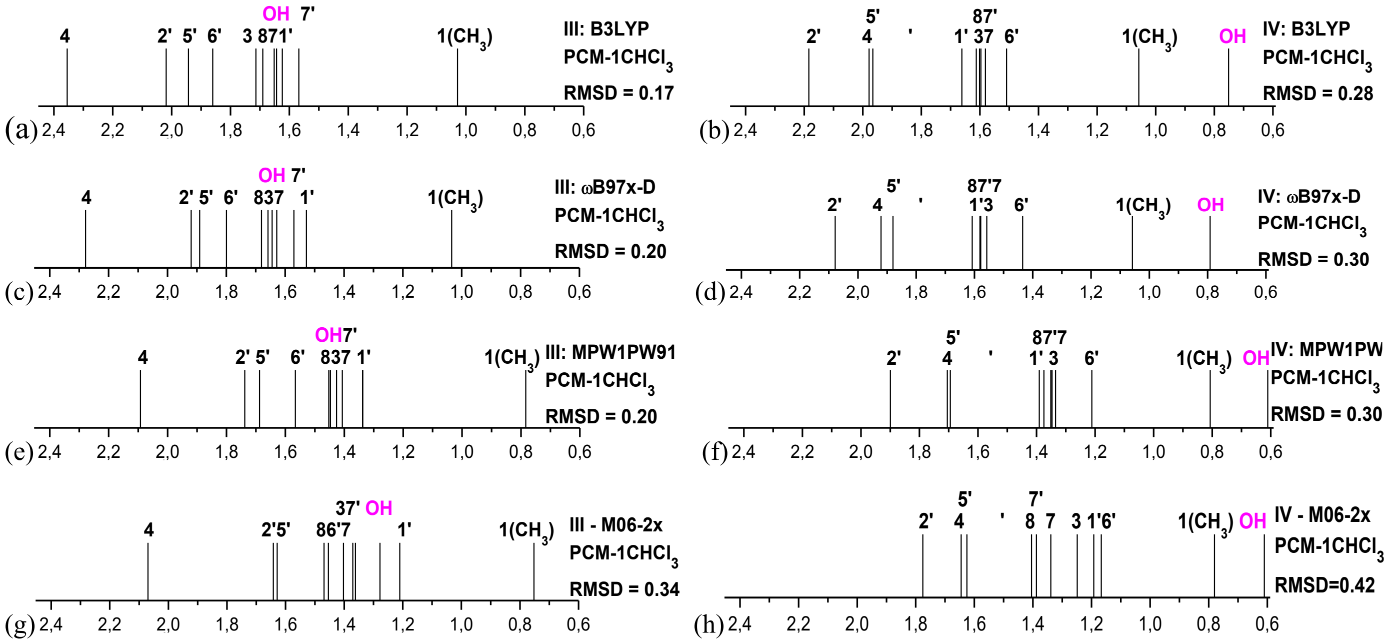

Figure S3. A comparative theoretical (PCM-1CHCl$_3$) ^{1}H NMR spectra for α-bisabol (structure **III**) and its epimer form (structure **IV**), calculated using distinct DFT functionals (B3LYP, ωB97x-D, MPW1PW91 and M0602x). RMSD data (in ppm) are also included. All PCM-1CHCl$_3$ structures have CHCl$_3$ at the same fixed position.

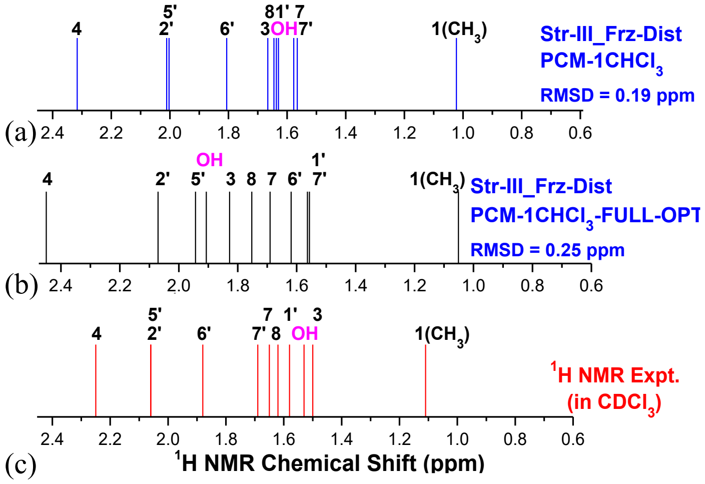

Figure S4. Theoretical (B3LYP/6-31G(d,p)-PCM-1CHCl$_3$) ^{1}H NMR spectra for two structures of α-bisabol (structure **III**). (a) Freezing the position of the CHCl$_3$ solvent molecule around the solute (Figure 5d and Figure S2a) and (b) fully optimizing the frozen-distance structure from (a). Experimental spectrum is also shown (c).

ωB97x-D/6-31G(d,p)-PCM-2CHCl$_3$ Optimized Structures
Charge = 0, Spin Multiplicity = 1

Str-I PCM-2CHCl$_3$

Atomic Number	X	Y	Z
6	-1.981274	1.859123	-0.524725
6	-0.640400	1.145616	-0.368794
6	0.432604	2.159771	0.067843
6	0.880340	3.147726	-1.020766
6	1.635403	4.323101	-0.457047
6	1.080995	5.389395	0.128570
6	-0.403851	5.572298	0.297065
6	1.920273	6.510105	0.677845
6	-0.249422	0.389586	-1.661234
6	-1.313579	-0.628202	-2.089258
6	-0.846216	-1.538298	-3.191832
6	0.421972	-1.657761	-3.589430
6	1.528145	-0.893282	-2.908257
6	1.103255	-0.323605	-1.558272
6	0.834781	-2.543496	-4.729167
1	-1.948616	2.569962	-1.353287
1	-2.216153	2.404513	0.392561
1	-2.790870	1.153693	-0.720878
1	0.025471	2.716420	0.917962
1	1.312424	1.626241	0.445387
1	1.507682	2.624869	-1.749304
1	0.004647	3.500211	-1.575770
1	2.720749	4.280271	-0.512121
1	-0.651752	5.688893	1.357206
1	-0.988464	4.737152	-0.093740
1	-0.734467	6.488589	-0.203566
1	1.750863	6.633573	1.753492
1	1.649683	7.461934	0.208044
1	2.985685	6.335973	0.513527
1	-0.166554	1.143990	-2.454870
1	-2.222684	-0.114646	-2.418445
1	-1.610481	-1.226549	-1.216887
1	-1.613920	-2.120356	-3.697115
1	2.395150	-1.551824	-2.776775
1	1.865782	-0.084144	-3.569992
1	1.019846	-1.151238	-0.841471
1	1.883624	0.343757	-1.181456
1	1.533308	-3.316020	-4.389874
1	1.358298	-1.964278	-5.497559
1	-0.023839	-3.033157	-5.192999
8	-0.842075	0.223296	0.713886
1	-0.023020	-0.261409	0.863685
6	-3.559796	-1.088753	1.095818
1	-2.590120	-0.631897	0.882633
17	-4.574494	-0.986934	-0.360640
17	-4.297766	-0.190252	2.438369
17	-3.279278	-2.784446	1.543677
6	3.228105	-2.225029	1.580443
1	2.543177	-2.999555	1.909201
17	4.704105	-2.344223	2.553827
17	2.428945	-0.655411	1.845218
17	3.567496	-2.487623	-0.138185

Str-II PCM-2CHCl$_3$

Atomic Number	X	Y	Z
6	0.914794	-1.367874	-1.892074
6	1.163074	-0.536609	-0.638166
6	1.266978	0.944288	-1.041238
6	1.585458	1.925045	0.094829
6	1.512578	3.352563	-0.374755
6	2.499849	4.041718	-0.955411
6	3.873914	3.486098	-1.216347
6	2.298328	5.458141	-1.419944
6	2.385549	-1.009628	0.175881
6	2.270397	-2.458196	0.659888
6	3.501835	-2.887249	1.411668
6	4.678382	-2.260419	1.336219
6	4.877915	-1.052476	0.453117
6	3.730935	-0.834198	-0.533407
6	5.879818	-2.698195	2.123573
1	1.791195	-1.351271	-2.543279
1	0.067221	-0.952717	-2.444863
1	0.682383	-2.406459	-1.645671
1	2.023726	1.058863	-1.825046
1	0.307647	1.215499	-1.495055
1	0.870496	1.785505	0.914767
1	2.575756	1.709885	0.509061
1	0.547605	3.844994	-0.260861
1	4.017730	2.482912	-0.811362
1	4.639969	4.141611	-0.789179
1	4.064263	3.444004	-2.294050
1	3.007361	6.132358	-0.927459
1	1.286028	5.810582	-1.213384
1	2.480883	5.544293	-2.496979
1	2.413310	-0.380913	1.078344
1	2.117995	-3.130796	-0.194621
1	1.382148	-2.564620	1.288011
1	3.412206	-3.764085	2.048783
1	4.993474	-0.165627	1.091491
1	5.827877	-1.154428	-0.084347
1	3.824456	0.157107	-0.986666
1	3.800522	-1.566576	-1.346105
1	6.255300	-1.877006	2.744202
1	6.699133	-2.985820	1.455744
1	5.650692	-3.543896	2.775313
8	-0.013237	-0.719642	0.166145
1	0.057864	-0.192671	0.969190
6	-2.989459	1.265718	1.177215
1	-3.383188	0.444907	0.584159
17	-2.031802	0.554268	2.497857
17	-4.362211	2.186836	1.817161
17	-1.977541	2.265671	0.124485
6	-2.455436	-2.004412	-0.958815
1	-1.526574	-1.681401	-0.483912
17	-2.160217	-3.497794	-1.862362
17	-2.967306	-0.686757	-2.039796
17	-3.662749	-2.262399	0.323904

Str-III PCM-2CHCl$_3$

Atomic Number	X	Y	Z
6	-0.585982	-2.647058	-1.459617
6	-0.975665	-1.783576	-0.261245
6	-0.602837	-2.577369	1.012489
6	-0.663392	-1.870786	2.375297
6	0.337136	-0.750532	2.491519
6	0.169920	0.493793	2.960809
6	-1.119781	1.046979	3.503505
6	1.329280	1.453560	3.014300
6	-2.482566	-1.393121	-0.290687
6	-3.059489	-1.248205	-1.705696
6	-4.471586	-0.726774	-1.696757
6	-5.059839	-0.152996	-0.644934
6	-4.302682	0.074472	0.639567
6	-2.797422	-0.087474	0.452024
6	-6.488871	0.305346	-0.669381
1	-1.217439	-3.537105	-1.511167
1	0.453979	-2.974263	-1.371416
1	-0.686821	-2.090133	-2.391821
1	-1.244176	-3.463185	1.040011
1	0.421728	-2.945967	0.880958
1	-1.679719	-1.541449	2.601166
1	-0.416482	-2.625217	3.131381
1	1.341841	-1.019527	2.159254
1	-0.951276	1.466348	4.499840
1	-1.914649	0.304463	3.580678
1	-1.475502	1.868036	2.871308
1	1.574304	1.694534	4.054499
1	1.075143	2.397933	2.522767
1	2.219733	1.044010	2.533052
1	-3.029065	-2.206783	0.206782
1	-3.045046	-2.210646	-2.226678
1	-2.415505	-0.575434	-2.290061
1	-5.041673	-0.847583	-2.615647
1	-4.527584	1.078997	1.017171
1	-4.669350	-0.622957	1.405908
1	-2.418646	0.744954	-0.149907
1	-2.297950	-0.023732	1.419466
1	-6.555533	1.384043	-0.489151
1	-7.065617	-0.181092	0.125080
1	-6.966844	0.083739	-1.625820
8	-0.158870	-0.601330	-0.372924
1	-0.217753	-0.137215	0.475976
6	0.947552	2.394553	-0.748421
1	1.165397	1.337768	-0.611068
17	0.710216	2.669679	-2.484480
17	2.316562	3.337736	-0.131608
17	-0.532836	2.771085	0.158817
6	2.776669	-1.428761	-0.456461
1	1.697820	-1.268951	-0.379618
17	3.218256	-2.864346	0.488905
17	3.553481	0.029886	0.198244
17	3.172848	-1.646113	-2.169725

Str-IV PCM-2CHCl$_3$

Atomic Number	X	Y	Z
6	-1.396387	-2.171253	-1.389278
6	-1.434603	-1.118340	-0.286853
6	-0.961951	-1.755171	1.031585
6	-0.993454	-0.824462	2.255380
6	-0.019869	-1.237178	3.329369
6	1.298824	-1.021761	3.283666
6	1.986773	-0.325141	2.138149
6	2.208426	-1.468517	4.394199
6	-2.802697	-0.419531	-0.155182
6	-3.227114	0.322818	-1.428175
6	-4.596104	0.927446	-1.283952
6	-5.475636	0.577931	-0.342512
6	-5.172437	-0.506692	0.662611
6	-3.942265	-1.336319	0.294900
6	-6.814717	1.242608	-0.206614
1	-2.180264	-2.916274	-1.236522
1	-0.437863	-2.698272	-1.382305
1	-1.527504	-1.716425	-2.371538
1	-1.543283	-2.659815	1.236286
1	0.067607	-2.089725	0.865188
1	-0.758118	0.199349	1.944084
1	-2.007388	-0.785172	2.664278
1	-0.423334	-1.760032	4.193485
1	1.322866	-0.178168	1.283130
1	2.846412	-0.909872	1.795977
1	2.373718	0.652922	2.448467
1	2.954324	-2.180204	4.023908
1	1.650491	-1.944131	5.203432
1	2.761836	-0.619226	4.808615
1	-2.684633	0.344736	0.629673
1	-3.218956	-0.364730	-2.283438
1	-2.496467	1.101334	-1.665974
1	-4.870493	1.701826	-1.996718
1	-5.028973	-0.044713	1.648800
1	-6.048262	-1.156997	0.764765
1	-3.644187	-1.949213	1.150284
1	-4.194923	-2.023535	-0.520536
1	-6.924591	1.692596	0.786650
1	-7.625447	0.513846	-0.312567
1	-6.951955	2.026615	-0.954629
8	-0.466641	-0.118420	-0.680252
1	-0.628542	0.673143	-0.153111
6	1.625171	2.476570	-0.053367
1	1.706527	1.405021	-0.216048
17	1.378859	3.245496	-1.627696
17	3.128329	3.016637	0.707494
17	0.230448	2.760023	1.018046
6	2.175564	-1.476423	-1.440027
1	1.251408	-1.004033	-1.097985
17	3.490861	-0.295684	-1.228863
17	1.964575	-1.897752	-3.150138
17	2.468575	-2.927720	-0.462197

Str-V PCM-2CHCl$_3$

Atomic Number	X	Y	Z

6	-0.416808	1.763409	2.352900
6	-0.825767	1.648646	0.888345
6	-0.373117	2.880687	0.085628
6	1.095661	3.279283	0.317011
6	1.687977	4.022228	-0.850544
6	2.199711	3.448674	-1.945051
6	2.261886	1.957050	-2.148613
6	2.760206	4.267936	-3.075494
6	-2.355810	1.423661	0.763879
6	-2.815304	0.190359	1.555657
6	-4.223145	-0.234797	1.244251
6	-4.948712	0.230387	0.225443
6	-4.369580	1.225342	-0.747499
6	-2.844817	1.297645	-0.684771
6	-6.372787	-0.181837	-0.013130
1	-0.923044	2.612102	2.818630
1	0.662038	1.906764	2.443806
1	-0.671656	0.856662	2.903907
1	-0.300500	0.398919	-0.545902
1	-0.497955	2.657164	-0.980178
1	-1.039422	3.722963	0.302149
1	1.175784	3.887779	1.221962
1	1.671955	2.369005	0.507130
1	1.670948	5.109651	-0.804568
1	1.705888	1.673061	-3.049551
1	1.854403	1.396348	-1.305211
1	3.297218	1.634893	-2.306357
1	2.250132	4.037514	-4.017742
1	3.821386	4.042107	-3.231672
1	2.660218	5.338877	-2.884760
1	-2.834956	2.312227	1.197521
1	-2.743565	0.387109	2.630443
1	-2.119850	-0.635817	1.353222
1	-4.669657	-0.961089	1.920609
1	-4.688797	0.967344	-1.763685
1	-4.798964	2.215881	-0.545152
1	-2.430412	0.380304	-1.124297
1	-2.501725	2.136441	-1.294995
1	-6.484706	-0.650626	-0.997211
1	-7.034613	0.691137	-0.004668
1	-6.722978	-0.885093	0.745344
8	-0.117006	0.502582	0.393863
6	0.411962	-2.822750	-1.513774
1	0.875481	-3.379915	-0.705985
17	1.405495	-1.377562	-1.795112
17	-1.224773	-2.372983	-0.993362
17	0.367183	-3.856662	-2.951960
6	1.923800	-1.009968	1.923003
1	0.985314	-0.730603	1.442134
17	1.750412	-0.817343	3.678825
17	3.164677	0.081278	1.281604
17	2.301473	-2.705405	1.524754

Str-VI PCM-2CHCl$_3$

Atomic Number	X	Y	Z
6	-1.226623	-2.795895	-1.125285
6	-1.237778	-1.612159	-0.167061

6	-0.712464	-1.992382	1.226367
6	0.638402	-2.724090	1.220332
6	1.399450	-2.562117	2.509922
6	2.067228	-1.461270	2.871566
6	2.168777	-0.227608	2.013726
6	2.790731	-1.375371	4.187017
6	-2.637093	-0.963326	-0.028083
6	-3.020083	-0.081357	-1.221291
6	-4.446118	0.393196	-1.136865
6	-5.364126	-0.109097	-0.307314
6	-5.046835	-1.267310	0.606838
6	-3.756765	-1.981014	0.213482
6	-6.760912	0.435689	-0.218724
1	-1.742456	-3.652316	-0.686531
1	-0.195405	-3.086917	-1.338160
1	-1.711671	-2.540931	-2.070010
1	-0.364701	0.132633	-0.190581
1	-0.613145	-1.060508	1.797599
1	-1.460843	-2.593716	1.752175
1	0.480028	-3.785257	1.010642
1	1.243926	-2.341981	0.393838
1	1.378575	-3.397103	3.207394
1	1.592938	-0.299862	1.089284
1	3.212421	-0.027672	1.745911
1	1.810220	0.651461	2.560922
1	3.858211	-1.185500	4.028812
1	2.686309	-2.295495	4.765147
1	2.410217	-0.542392	4.787889
1	-2.596510	-0.307306	0.856220
1	-2.860634	-0.627681	-2.160848
1	-2.355741	0.787245	-1.271477
1	-4.724068	1.214060	-1.794514
1	-4.973972	-0.899152	1.639004
1	-5.884886	-1.973428	0.599923
1	-3.475927	-2.704255	0.984218
1	-3.924842	-2.544448	-0.711927
1	-6.978310	0.772295	0.800868
1	-7.497528	-0.339433	-0.456478
1	-6.913267	1.276261	-0.898706
8	-0.328467	-0.655854	-0.745107
6	0.489307	2.974939	0.195586
1	0.806627	2.323877	-0.615303
17	-0.687824	2.066883	1.182432
17	-0.261204	4.412069	-0.508117
17	1.922494	3.384445	1.146198
6	2.550714	-0.792143	-1.727283
1	1.541719	-0.945066	-1.334869
17	2.510623	-1.108027	-3.471658
17	3.673721	-1.890623	-0.905485
17	2.989578	0.905095	-1.396734

Str-VII PCM-2$CHCl_3$

Atomic Number	X	Y	Z
6	1.427012	-1.569684	-2.299804
6	1.693628	-0.666034	-1.100878

6	2.434070	0.610881	-1.587380
6	1.982341	1.937358	-0.955428
6	2.734891	3.091834	-1.555645
6	3.794653	3.726420	-1.044839
6	4.422022	3.415434	0.287062
6	4.462454	4.846552	-1.796064
6	2.504362	-1.422592	-0.015782
6	1.783212	-2.695410	0.454163
6	2.377198	-3.298718	1.696631
6	3.284243	-2.701687	2.472410
6	3.766043	-1.304842	2.176090
6	2.860800	-0.555717	1.199463
6	3.889099	-3.368790	3.673907
1	2.371552	-1.885876	-2.748731
1	0.852240	-1.023711	-3.052276
1	0.857270	-2.456653	-2.014824
1	0.488727	0.178008	0.208488
1	3.511138	0.488537	-1.426344
1	2.298635	0.704449	-2.670636
1	0.911333	2.068648	-1.141094
1	2.109744	1.914645	0.131081
1	2.389822	3.402748	-2.541398
1	5.482642	3.173062	0.155181
1	3.946847	2.580656	0.805336
1	4.381616	4.294036	0.940845
1	5.523101	4.625682	-1.961632
1	4.422125	5.780204	-1.223453
1	3.991148	5.018112	-2.765776
1	3.451075	-1.716212	-0.491423
1	1.803767	-3.452488	-0.337666
1	0.720077	-2.466062	0.611883
1	2.045146	-4.304033	1.949638
1	3.846268	-0.744233	3.115276
1	4.787438	-1.356915	1.773594
1	1.940216	-0.264088	1.725286
1	3.363478	0.364606	0.889429
1	3.663895	-2.805140	4.587445
1	4.981983	-3.404733	3.588876
1	3.519962	-4.390028	3.795242
8	0.390807	-0.334411	-0.600004
6	-3.618490	1.508352	1.464240
1	-4.062263	0.670377	0.936800
17	-4.210302	3.006727	0.724957
17	-1.853938	1.369046	1.282378
17	-4.092574	1.401267	3.171050
6	-2.119223	-1.602570	-1.668980
1	-1.314169	-0.936047	-1.349479
17	-1.855405	-3.159843	-0.857996
17	-2.040787	-1.784915	-3.431218
17	-3.681180	-0.896222	-1.188268

Str-VIII PCM-2$CHCl_3$

Atomic Number	X	Y	Z
6	1.883552	0.091002	1.993419
6	1.638350	0.162316	0.490923
6	2.881936	-0.310843	-0.299779
6	4.273474	0.001966	0.275518
6	5.358594	-0.471738	-0.655707

6	5.841073	-1.716048	-0.731892
6	5.379417	-2.851613	0.141330
6	6.901489	-2.087639	-1.731877
6	1.130064	1.545435	0.010019
6	-0.239969	1.907704	0.597383
6	-0.653395	3.322225	0.307507
6	0.159865	4.265511	-0.169476
6	1.617587	3.984244	-0.427669
6	2.112728	2.697953	0.233741
6	-0.311243	5.654585	-0.487212
1	2.641234	0.814069	2.300915
1	2.233579	-0.909185	2.260167
1	0.969990	0.289577	2.558130
1	0.345006	-0.740550	-0.683755
1	2.802933	-1.400362	-0.370332
1	2.823033	0.073596	-1.325400
1	4.389750	1.074526	0.451066
1	4.374026	-0.476607	1.254325
1	5.745081	0.265585	-1.357222
1	4.622231	-2.553435	0.868169
1	6.226598	-3.285125	0.683359
1	4.959168	-3.653421	-0.474876
1	7.789763	-2.481184	-1.225667
1	7.202323	-1.232502	-2.340355
1	6.544600	-2.879306	-2.399690
1	0.997160	1.449981	-1.080462
1	-0.248360	1.744330	1.683983
1	-0.999025	1.233616	0.189890
1	-1.696698	3.568618	0.494079
1	1.782879	3.934893	-1.512603
1	2.212083	4.835959	-0.077881
1	3.098002	2.450926	-0.167998
1	2.234187	2.862841	1.310785
1	-0.114485	5.899560	-1.536773
1	0.228515	6.394483	0.113289
1	-1.381330	5.770425	-0.301827
8	0.592195	-0.799763	0.247186
6	-2.923267	-0.947459	-1.813875
1	-2.742576	-1.247592	-0.785497
17	-3.103169	-2.417086	-2.783436
17	-1.516785	0.003046	-2.353919
17	-4.393997	0.034690	-1.846369
6	-1.845901	-1.018210	2.030516
1	-0.969165	-0.748087	1.433813
17	-1.395552	-0.974060	3.742440
17	-2.315841	-2.660703	1.527748
17	-3.159019	0.129767	1.683164

Str-IX PCM-2CHCl$_3$

Atomic Number	X	Y	Z
6	0.732805	0.861779	2.603650
6	1.128370	0.381977	1.208654
6	2.461157	-0.377196	1.357726
6	3.107563	-0.984893	0.104014
6	4.281986	-1.852533	0.474624
6	5.532980	-1.438424	0.697197
6	5.977527	-0.004509	0.591632

6	6.617902	-2.406909	1.083495
6	1.227227	1.549635	0.184476
6	0.264474	2.710307	0.468353
6	0.291272	3.755430	-0.613402
6	0.808616	3.567818	-1.828815
6	1.367898	2.229037	-2.238916
6	0.992568	1.112979	-1.268482
6	0.881566	4.659665	-2.857067
1	1.465401	1.581463	2.975805
1	0.694464	0.011628	3.287390
1	-0.244777	1.347542	2.611889
1	0.409299	-1.035691	0.060991
1	3.188537	0.307922	1.807964
1	2.294232	-1.178512	2.085672
1	2.383862	-1.599967	-0.444931
1	3.418299	-0.189578	-0.580402
1	4.065357	-2.910286	0.612158
1	6.403353	0.327979	1.544301
1	5.168201	0.679119	0.328900
1	6.770730	0.096610	-0.157163
1	7.071984	-2.123813	2.039095
1	7.422441	-2.401680	0.339559
1	6.237141	-3.426510	1.170330
1	2.249523	1.946393	0.259419
1	0.510040	3.184968	1.422995
1	-0.757569	2.320445	0.582174
1	-0.127888	4.727816	-0.362982
1	1.008558	1.982301	-3.244809
1	2.460511	2.306391	-2.323071
1	-0.071160	0.875932	-1.388357
1	1.545379	0.209808	-1.532063
1	0.342475	4.379472	-3.768408
1	1.921836	4.840795	-3.150025
1	0.464845	5.596163	-2.480585
8	0.091123	-0.540240	0.823916
6	-2.719098	0.163356	1.822870
1	-1.751201	0.101765	1.320340
17	-3.173661	1.870866	1.989206
17	-2.540983	-0.605969	3.411349
17	-3.902793	-0.710981	0.824442
6	-1.724635	-1.737731	-1.750272
1	-1.719829	-1.336793	-0.739875
17	-2.876689	-3.078139	-1.806800
17	-0.072033	-2.305533	-2.106139
17	-2.194672	-0.431331	-2.847708

Str-X PCM-2CHCl$_3$

Atomic Number	X	Y	Z
6	2.144119	0.139890	1.720034
6	1.754122	0.015898	0.251534
6	2.731009	-0.902903	-0.503550
6	4.182089	-0.433917	-0.688543
6	5.078878	-1.566060	-1.121183
6	5.583168	-2.501792	-0.310345
6	5.332558	-2.540240	1.173916
6	6.446649	-3.617239	-0.834014
6	1.562479	1.393747	-0.437174

6	0.201929	2.026981	-0.117405
6	0.074209	3.425244	-0.656237
6	1.098636	4.161687	-1.091531
6	2.514506	3.646907	-1.017570
6	2.645380	2.430118	-0.104472
6	0.916787	5.535496	-1.668588
1	3.135045	0.583084	1.832333
1	2.155140	-0.852978	2.176735
1	1.430583	0.762810	2.266281
1	0.208285	-0.832158	-0.630368
1	2.738193	-1.855047	0.036821
1	2.305115	-1.111439	-1.493527
1	4.220157	0.365933	-1.433226
1	4.556098	-0.002802	0.246017
1	5.299006	-1.635776	-2.184475
1	4.695369	-1.725994	1.524423
1	6.278649	-2.498717	1.724234
1	4.851463	-3.484111	1.450734
1	7.424246	-3.617934	-0.338736
1	6.607604	-3.535127	-1.911081
1	5.990890	-4.591768	-0.626522
1	1.595146	1.217202	-1.523356
1	0.049956	2.042219	0.972591
1	-0.610587	1.416070	-0.522153
1	-0.931404	3.838216	-0.700384
1	2.862433	3.398004	-2.029480
1	3.172244	4.450586	-0.666793
1	3.650877	2.014747	-0.183643
1	2.512769	2.747677	0.936687
1	1.327438	5.587136	-2.682889
1	1.453544	6.283520	-1.075324
1	-0.136884	5.817886	-1.709653
8	0.500705	-0.693863	0.278168
6	-1.823117	0.083624	2.056468
1	-0.971396	-0.068599	1.385525
17	-2.553069	-1.513468	2.347013
17	-2.992429	1.164446	1.262208
17	-1.226761	0.799735	3.563866
6	-3.581310	-1.359462	-1.258352
1	-3.579462	-1.108396	-0.202580
17	-5.077454	-0.728976	-1.965575
17	-3.489157	-3.121517	-1.399960
17	-2.159290	-0.581467	-1.997050

****
